# Supplementary material for: Sesquiterpenoids from Inula britannica and Their Potential Effects against Triple-Negative Breast Cancer Cells
Source: Molecules. 2022 Aug 16;27(16):5230. doi: 10.3390/molecules27165230 (PMC9412570; doi:10.3390/molecules27165230)
Supplement: Supplementary file 1 [file molecules-27-05230-s001.zip › molecules-1868799-supplementary.pdf]

*Supplementary Materials*

# Sesquiterpenoids from *Inula britannica* and Their Potential Effects against Triple Negative Breast Cancer Cells

**Ruo-Yu Qi, Cong Guo, Xiao-Na Peng and Jiang-Jiang Tang ***

Shaanxi Key Laboratory of Natural Products & Chemical Biology, College of Chemistry & Pharmacy, Northwest A&F University, Taicheng Road, Yangling 712100, China

* Correspondence: tangjiang11@nwafu.edu.cn (J.-J.T.); Tel.: +86-298-709-2335

**Table S1.** Crystal data and structure refinement for **4**.

| | |
|---|---|
| Identification code | **4** |
| Empirical formula | $C_{15}H_{20}O_4$ |
| Formula weight | 264.31 |
| Temperature/K | 149.96(10) |
| Crystal system | orthorhombic |
| Space group | $P2_12_12_1$ |
| a/Å | 9.55914(9) |
| b/Å | 10.00802(10) |
| c/Å | 13.83442(15) |
| α/° | 90 |
| β/° | 90 |
| γ/° | 90 |
| Volume/Å$^3$ | 1323.51(2) |
| Z | 4 |
| $\rho_{calc}$g/cm$^3$ | 1.326 |
| μ/mm$^{-1}$ | 0.779 |
| F(000) | 568.0 |
| Crystal size/mm$^3$ | 0.22 × 0.16 × 0.05 |
| Radiation | Cu Kα (λ = 1.54184) |
| 2Θ range for data collection/° | 10.912 to 154.8 |
| Index ranges | $-9 \leq h \leq 11$, $-12 \leq k \leq 12$, $-17 \leq l \leq 16$ |
| Reflections collected | 7356 |
| Independent reflections | 2626 [$R_{int}$ = 0.0173, $R_{sigma}$ = 0.0153] |
| Data/restraints/parameters | 2626/0/183 |
| Goodness-of-fit on F$^2$ | 1.010 |
| Final R indexes [I>=2σ (I)] | $R_1$ = 0.0301, $wR_2$ = 0.1041 |
| Final R indexes [all data] | $R_1$ = 0.0303, $wR_2$ = 0.1044 |
| Largest diff. peak/hole / e Å$^{-3}$ | 0.19/-0.17 |
| Flack parameter | -0.04(5) |

**Spectral data for isolates 1–8:**

**Figure S1.** $^{1}$H, $^{13}$C NMR, HSQC, HMBC and $^{1}$H-$^{1}$H COSY ($CDCl_3$) spectra of **1**.

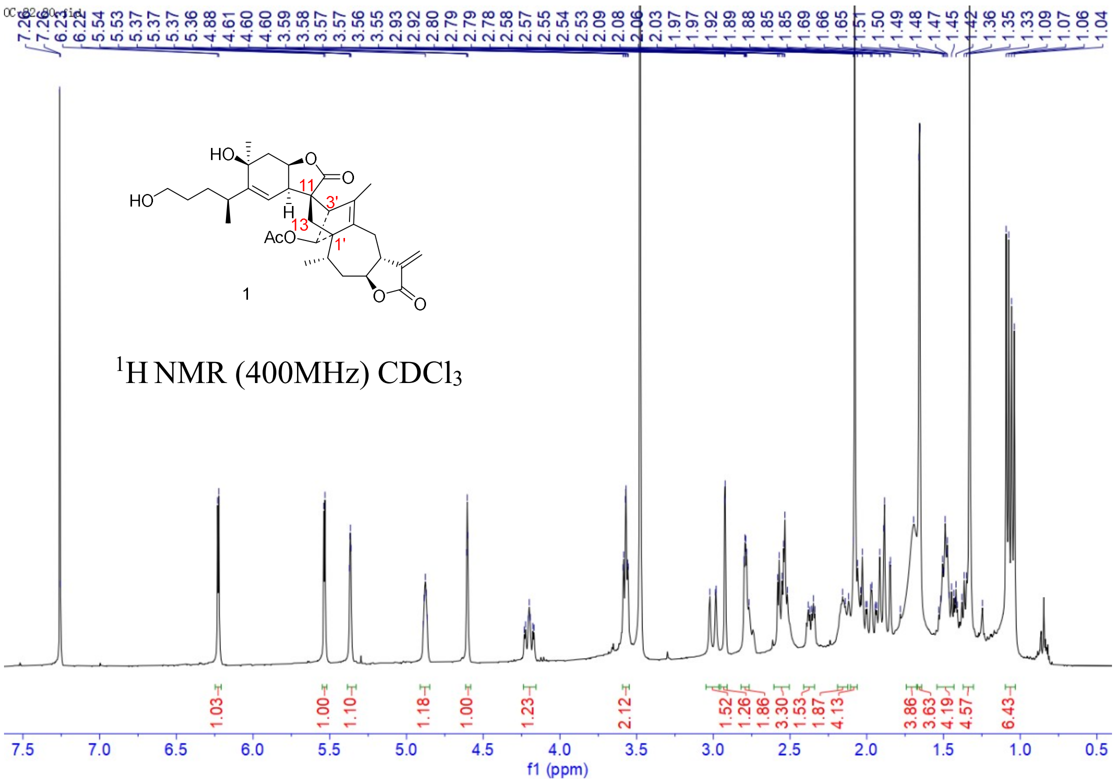


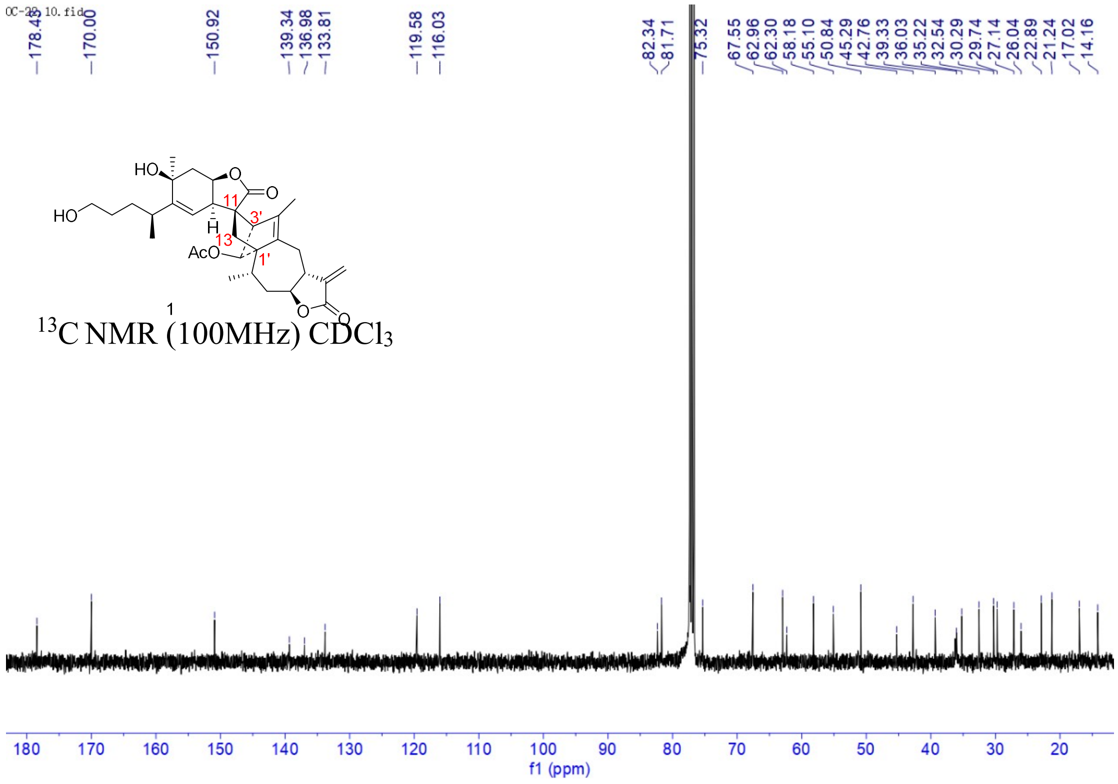

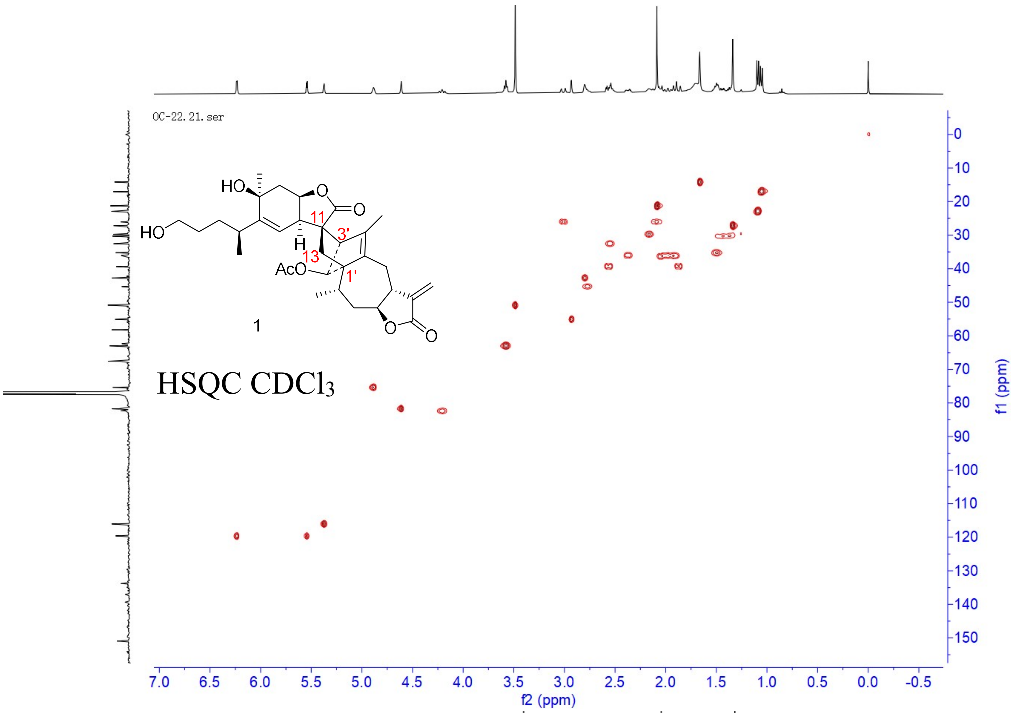
OC-22.21.ser
HO
HO
AcO
11
3'
13
1'
H
1
HSQC CDCl3
f2 (ppm)
f1 (ppm)

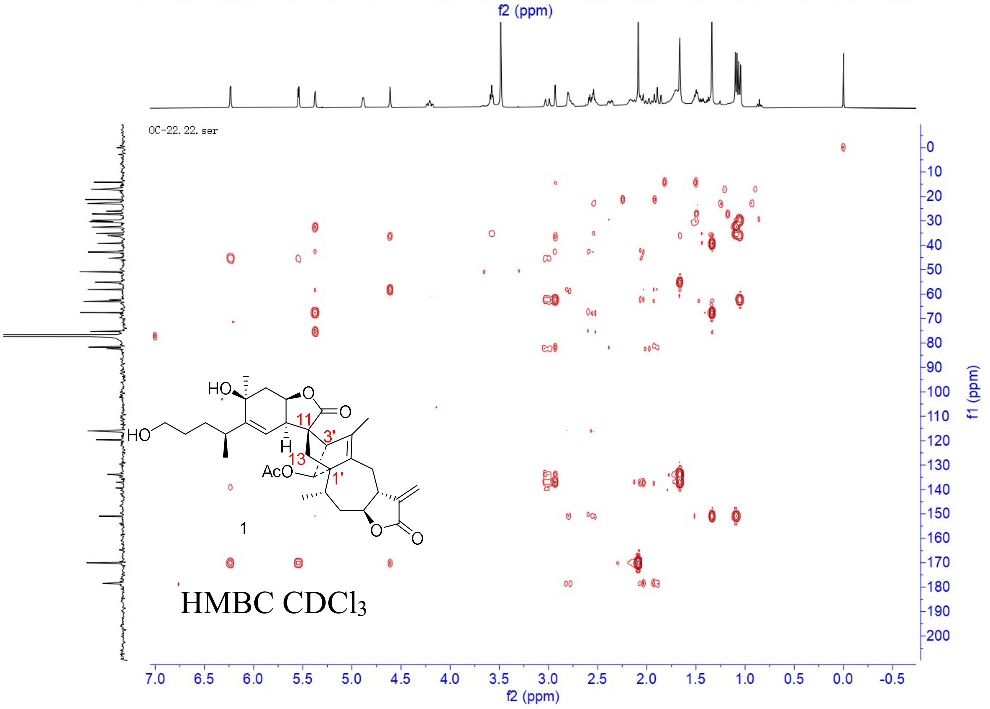
OC-22.22.ser
HO
HO
AcO
11
3'
13
1'
H
1
HMBC CDCl3
f2 (ppm)
f1 (ppm)

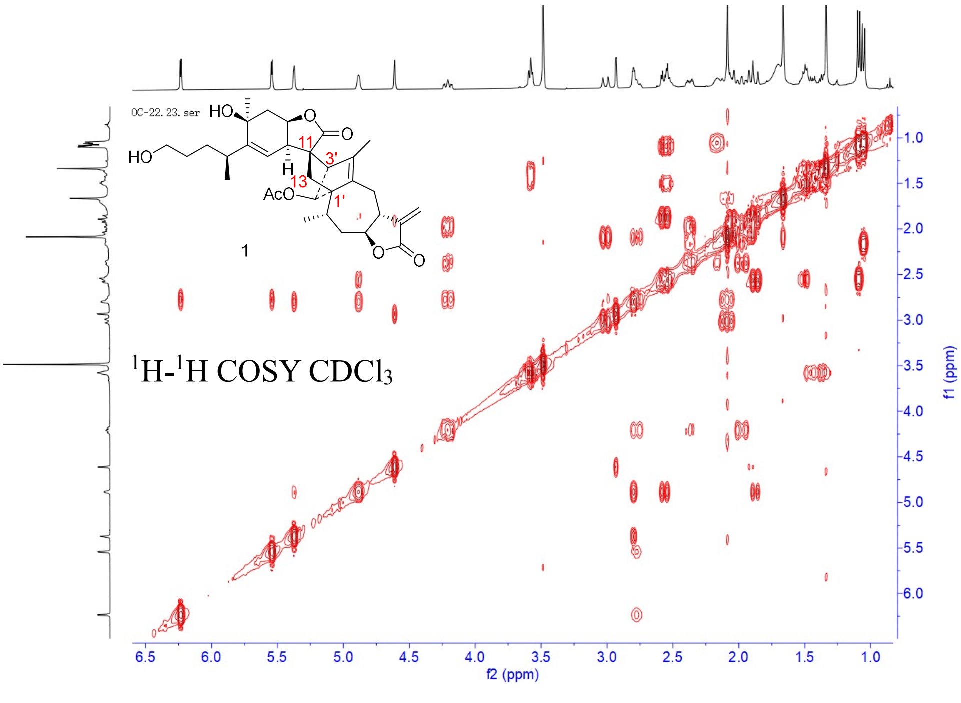
OC-22.23.ser
HO
HO
AcO
11
3'
13
1'
H
1
1H-1H COSY CDCl3
f2 (ppm)
f1 (ppm)

**Figure S2.** $^1$H ,$^{13}$C, DEPT, HSQC and $^1$H-$^1$H COSY NMR ($CDCl_3$) spectra of **2**.

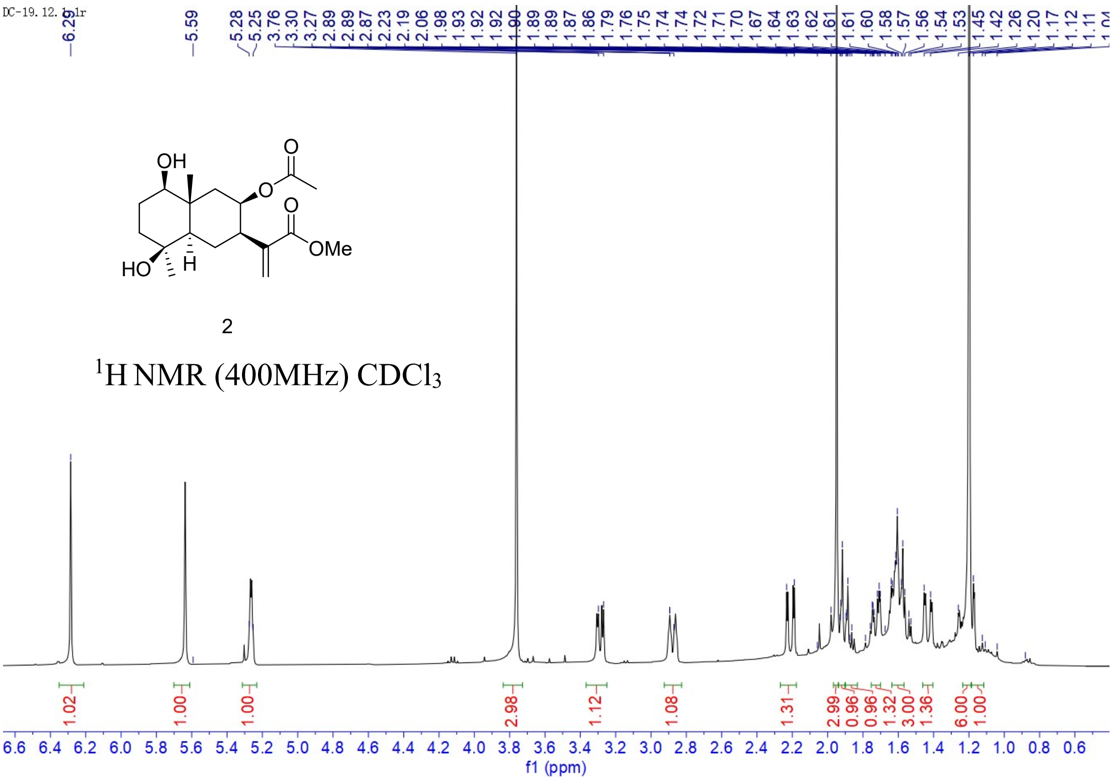


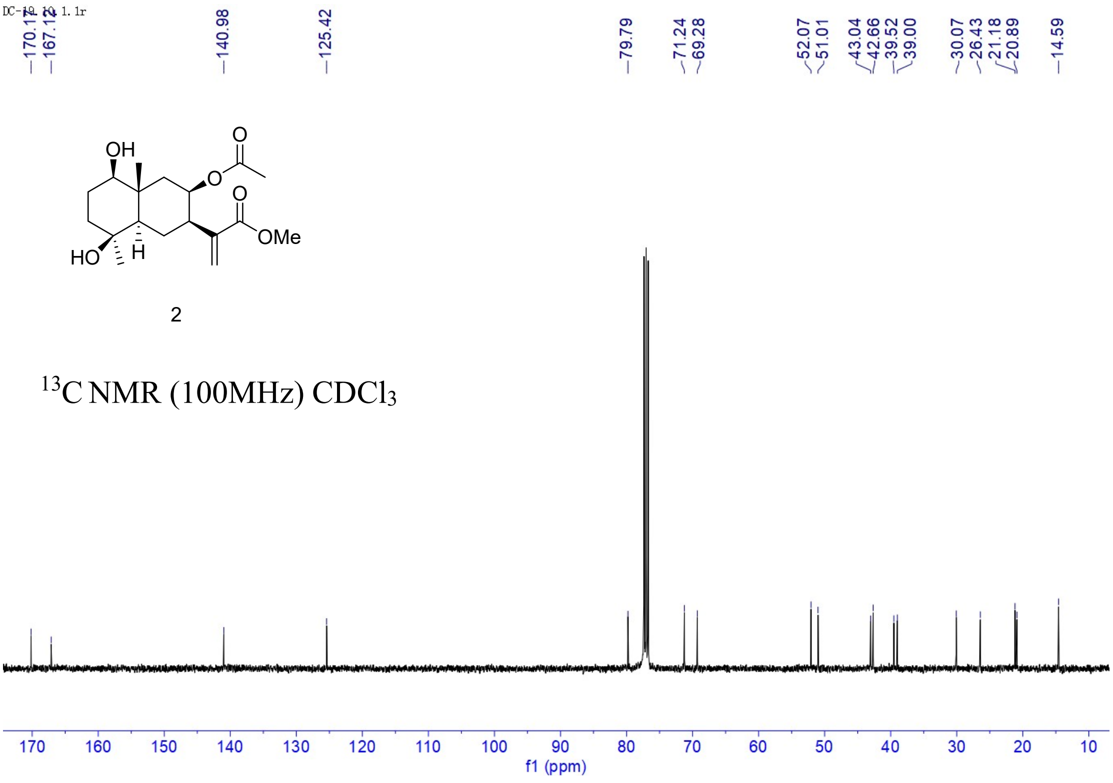

DC-19.11.fid

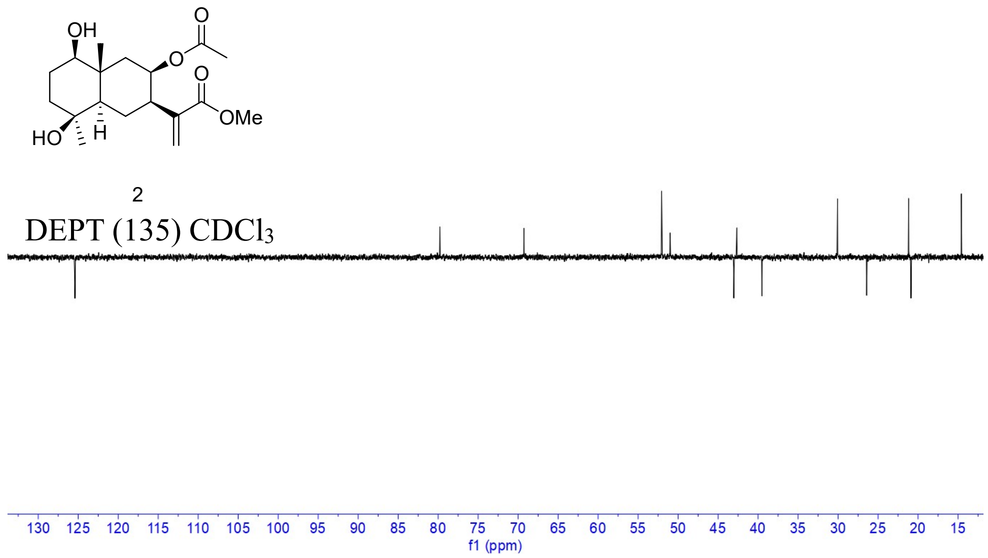

OH
O
O
OMe
HO
H
2
DEPT (135) CDCl3
f1 (ppm)


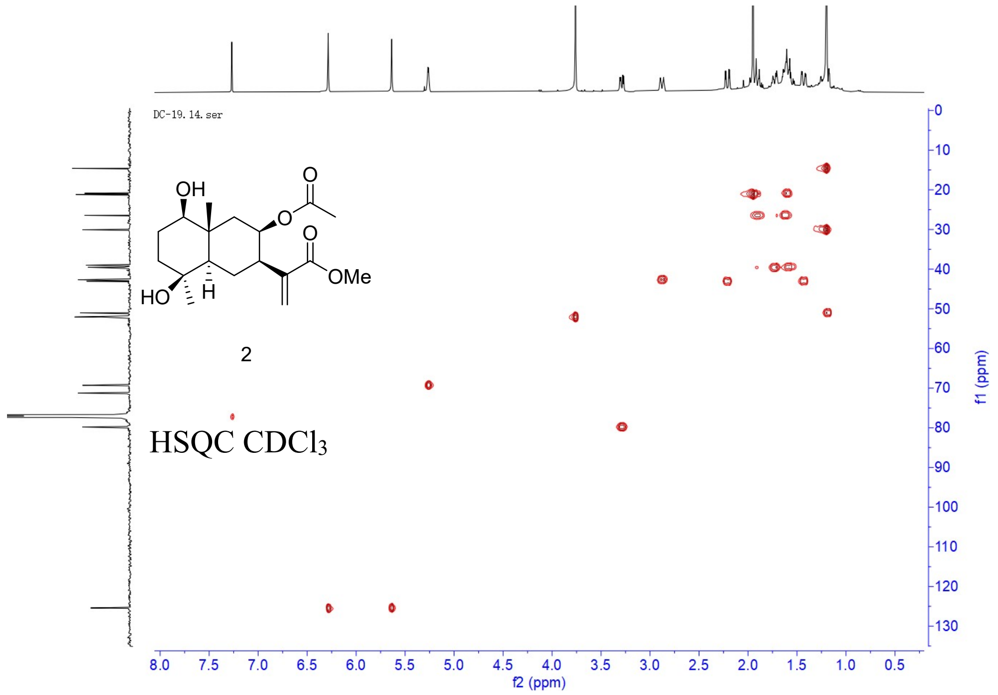

DC-19.14.ser
OH
O
O
OMe
HO
H
2
HSQC CDCl3
f2 (ppm)
f1 (ppm)


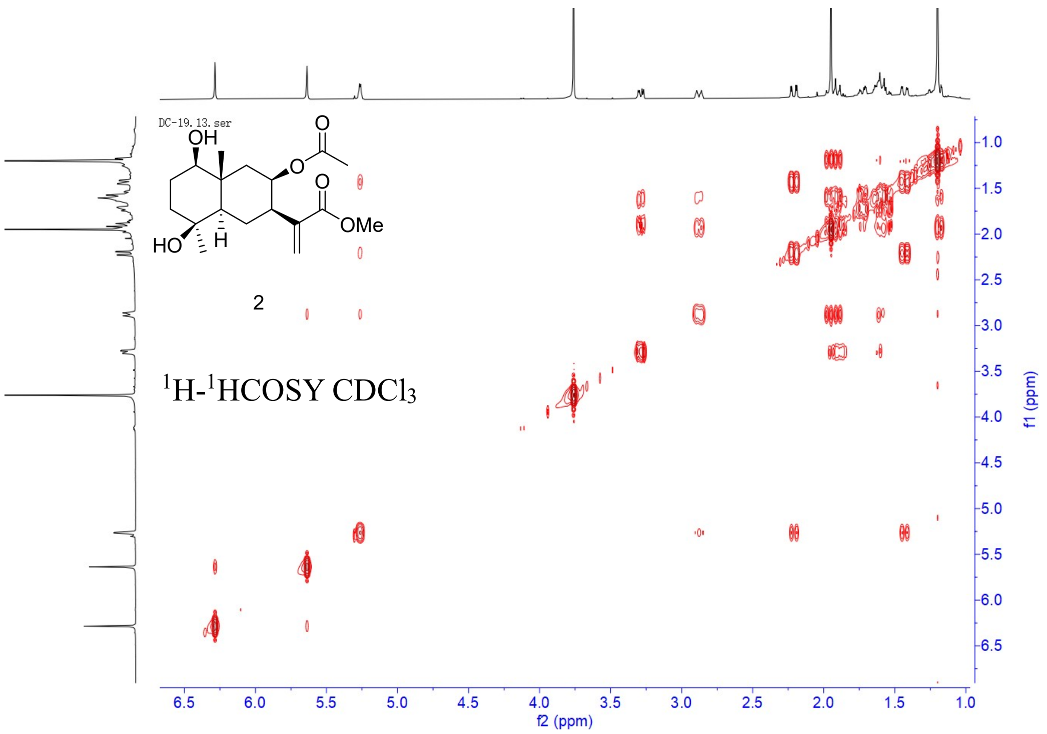

DC-19.13.ser
OH
O
O
OMe
HO
H
2
1H-1HCOSY CDCl3
f2 (ppm)
f1 (ppm)

**Figure S3.** $^{1}H$ ,$^{13}C$, DEPT, HSQC and HMBC NMR (MeOD) spectra of **3**

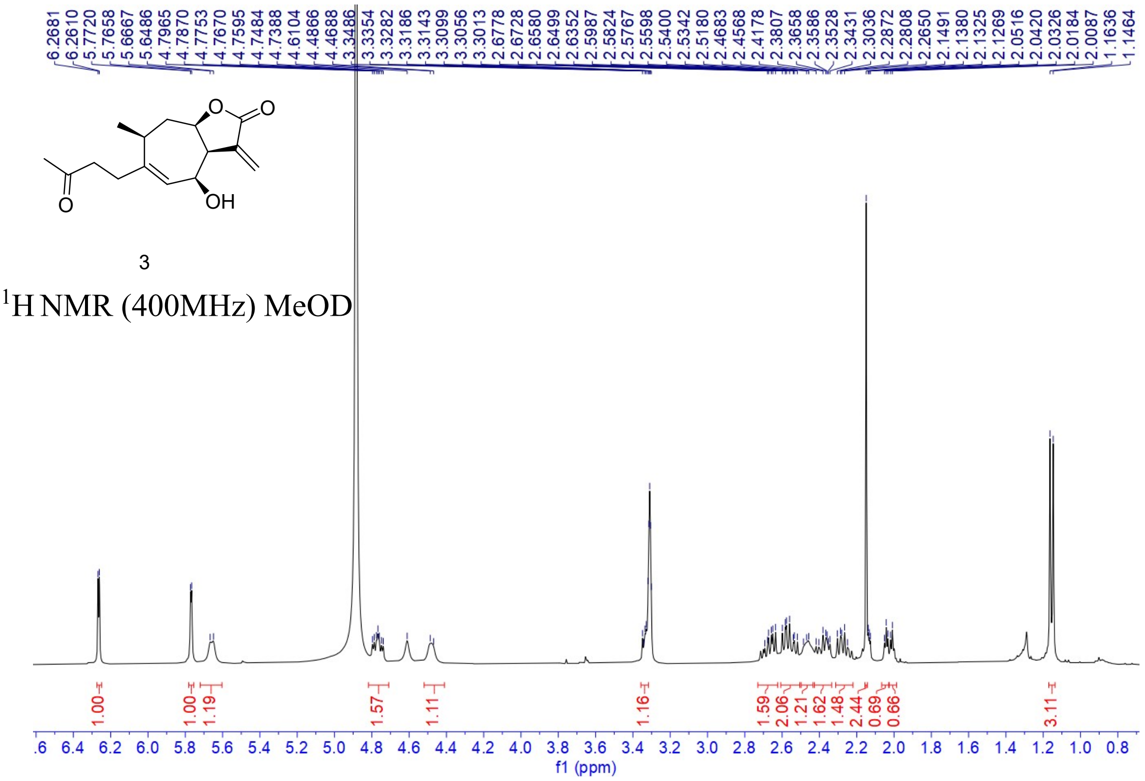


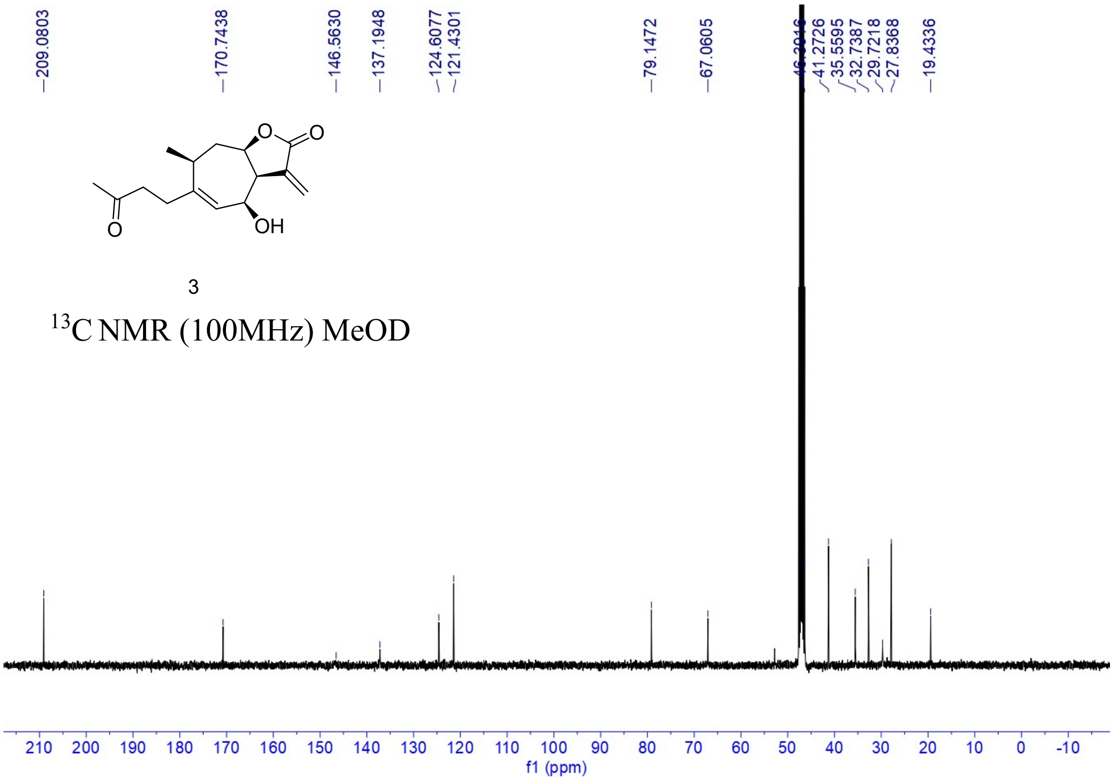

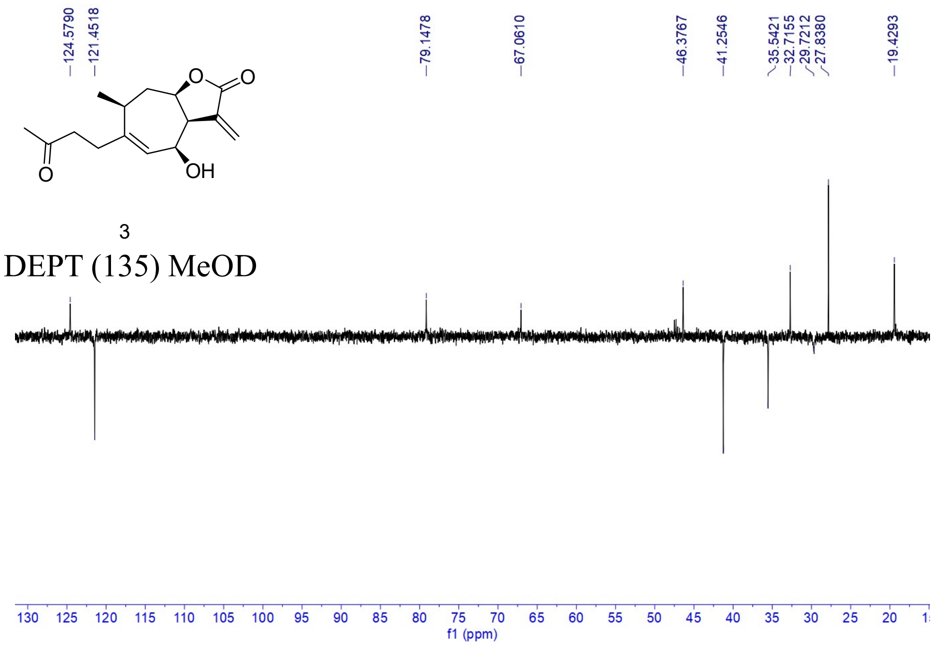
124.5790
121.4518
79.1478
67.0610
46.3767
41.2546
35.5421
32.7155
29.7212
27.8380
19.4293
OH
3
DEPT (135) MeOD
f1 (ppm)

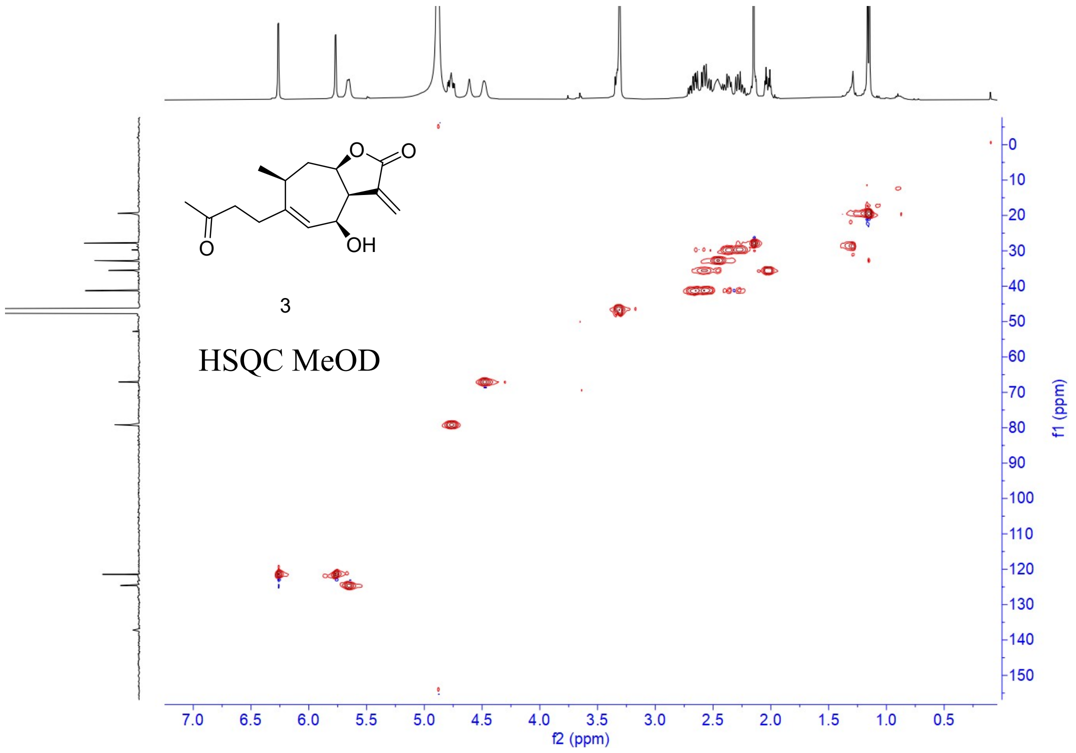
OH
3
HSQC MeOD
f1 (ppm)
f2 (ppm)

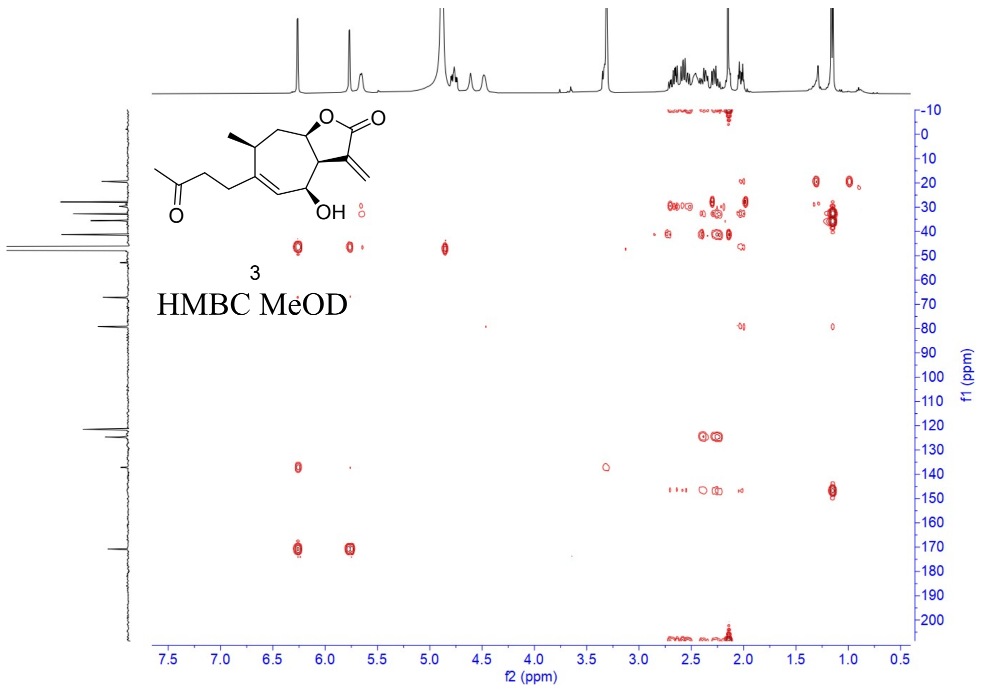
OH
3
HMBC MeOD
f1 (ppm)
f2 (ppm)

**Figure S4.** $^{1}H$ ,$^{13}C$, DEPT, HSQC, HMBC and $^{1}H$-$^{1}H$ COSY ($CDCl_3$) spectra of **4**

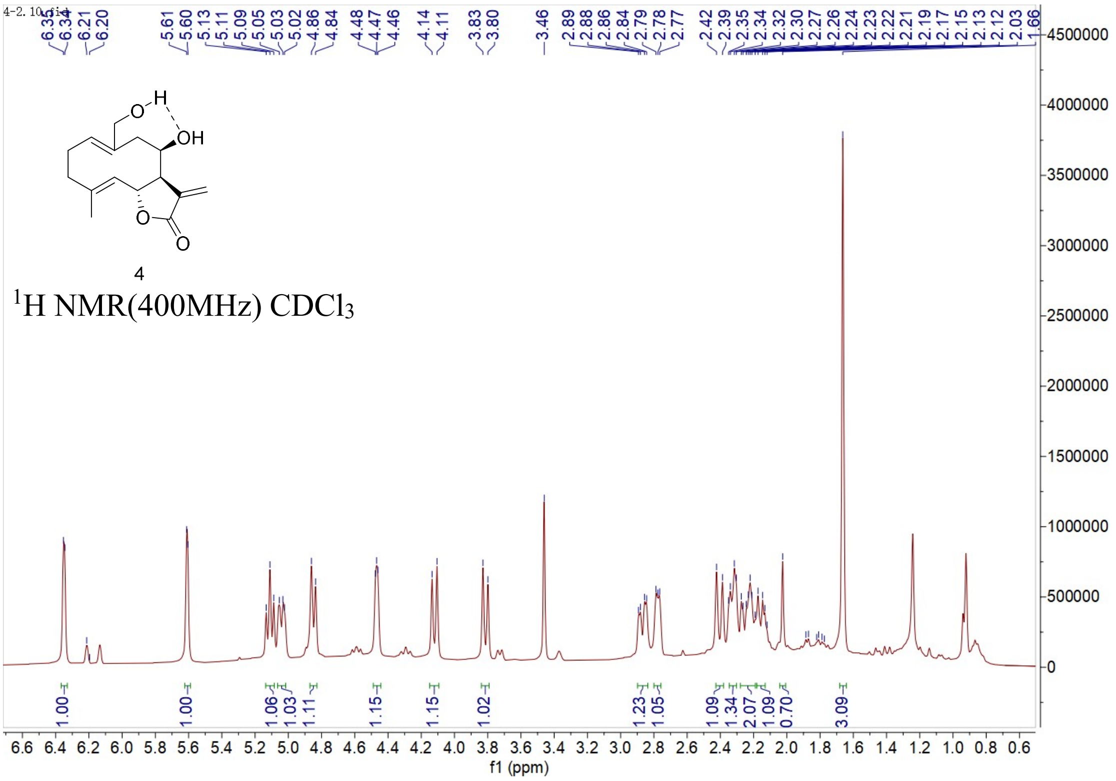


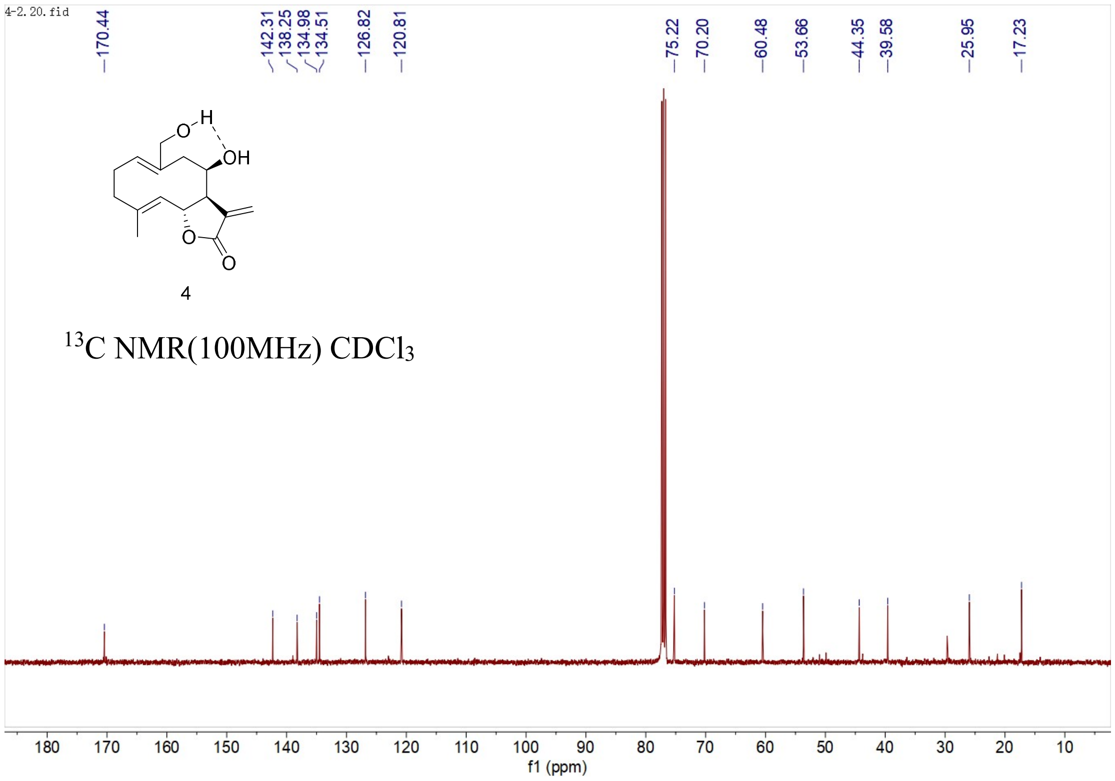

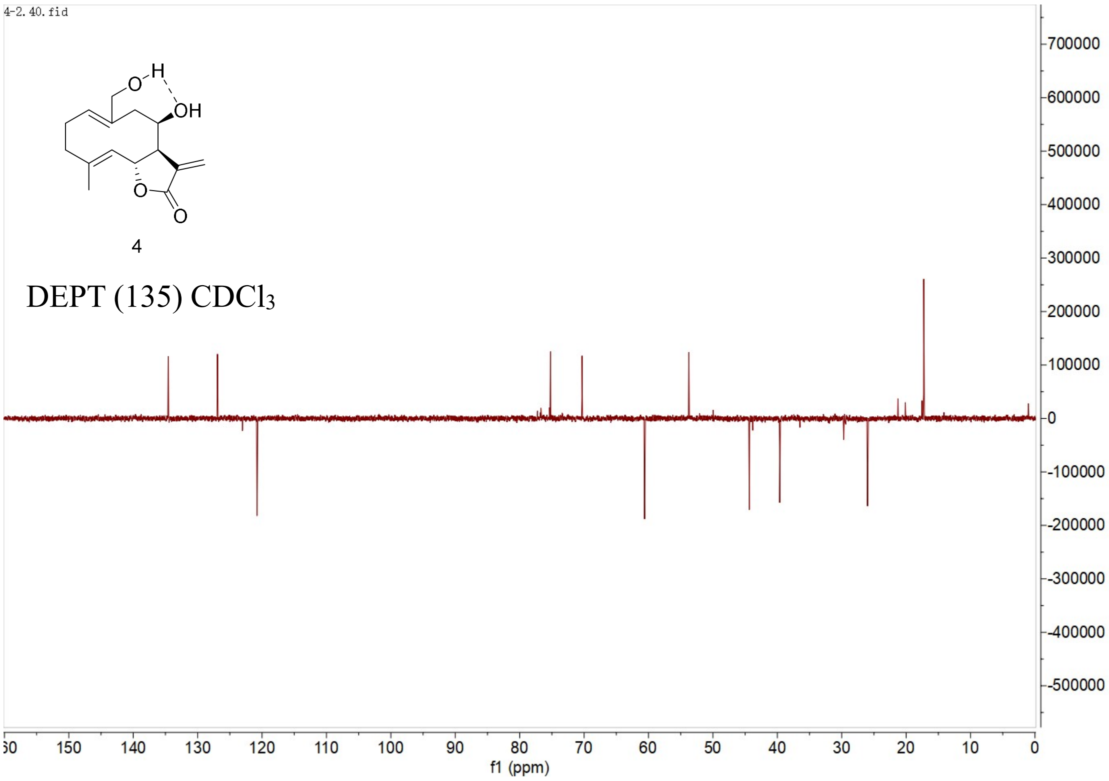
4-2.40.fid
4
DEPT (135) CDCl3
f1 (ppm)

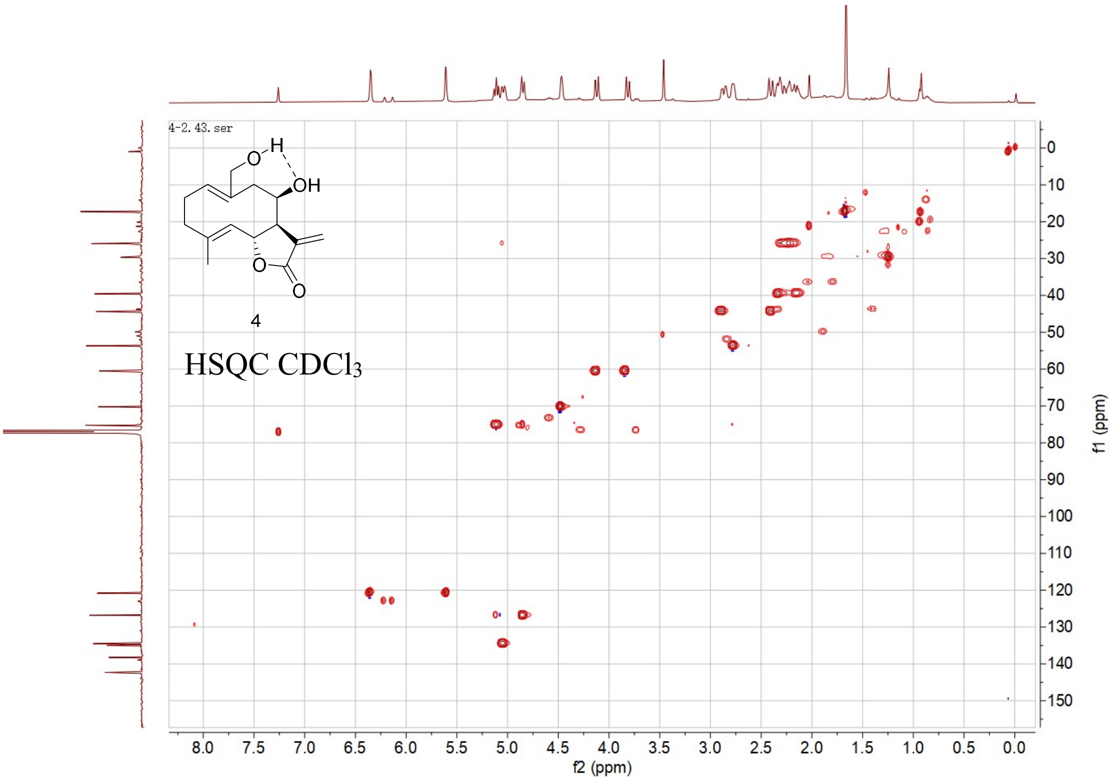
4-2.43.ser
4
HSQC CDCl3
f2 (ppm)
f1 (ppm)

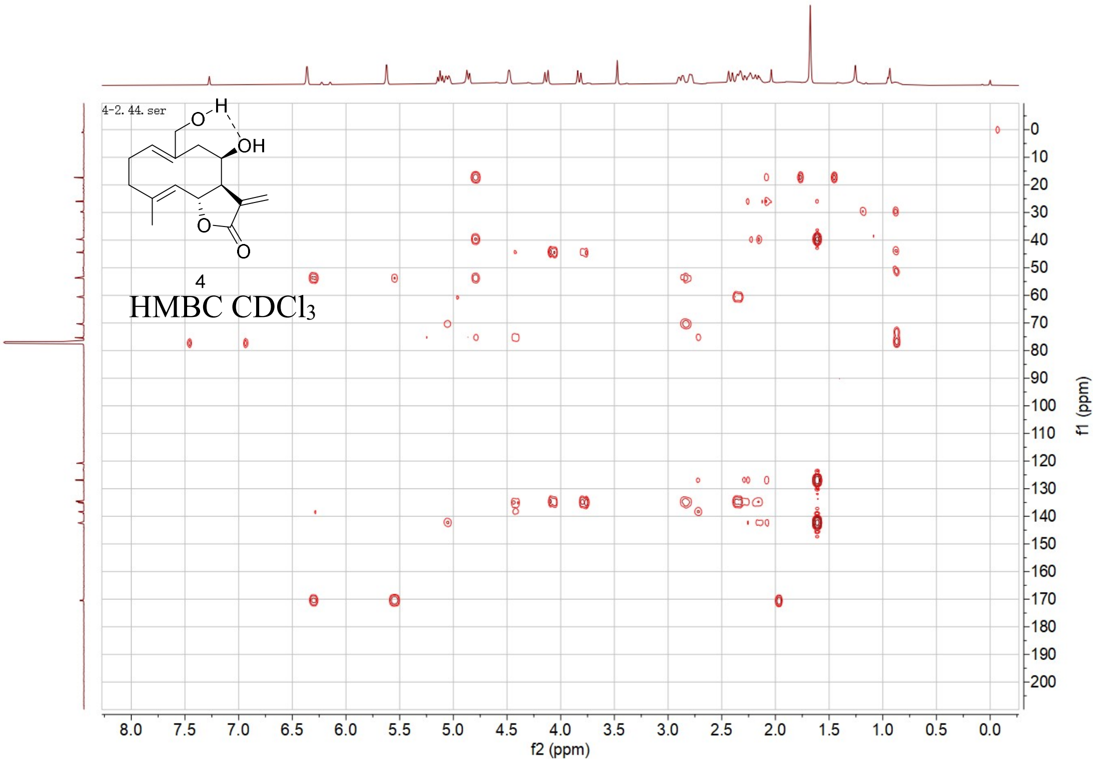
4-2.44.ser
4
HMBC CDCl3
f2 (ppm)
f1 (ppm)

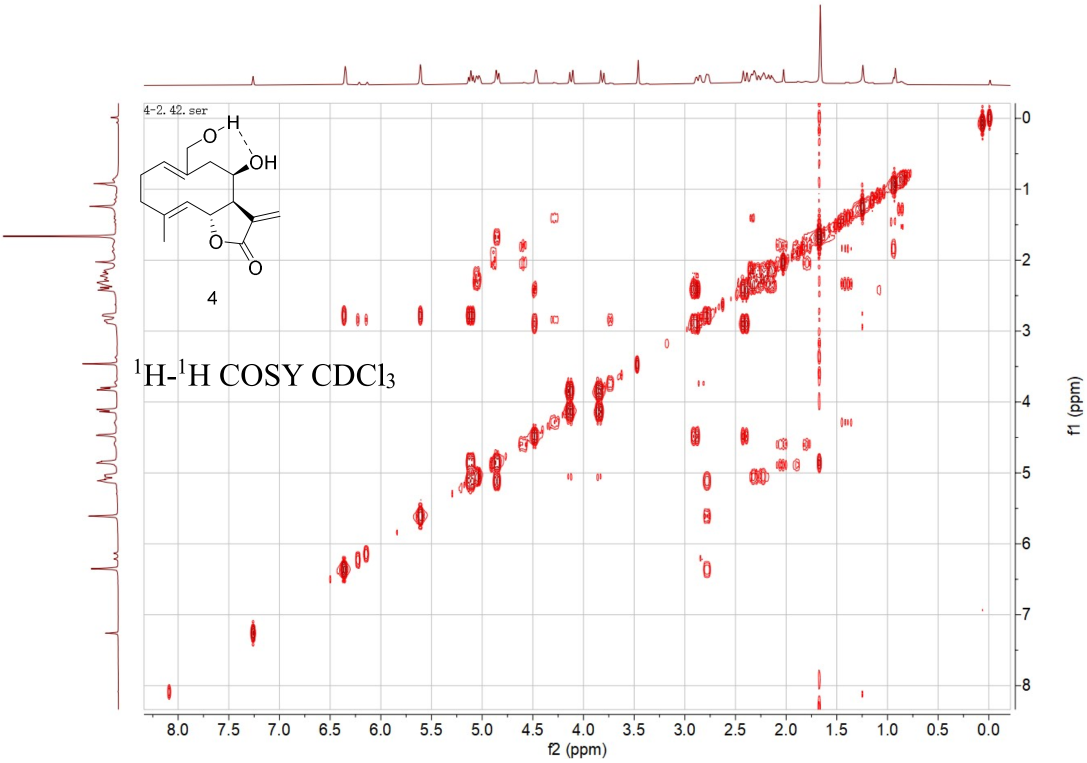
4-2.42.ser
4
1H-1H COSY CDCl3
f2 (ppm)
f1 (ppm)

**Figure S5.** $^1$H ,$^{13}$C, HSQC, HMBC and $^1$H-$^1$H COSY NMR (MeOD) spectra of **5**

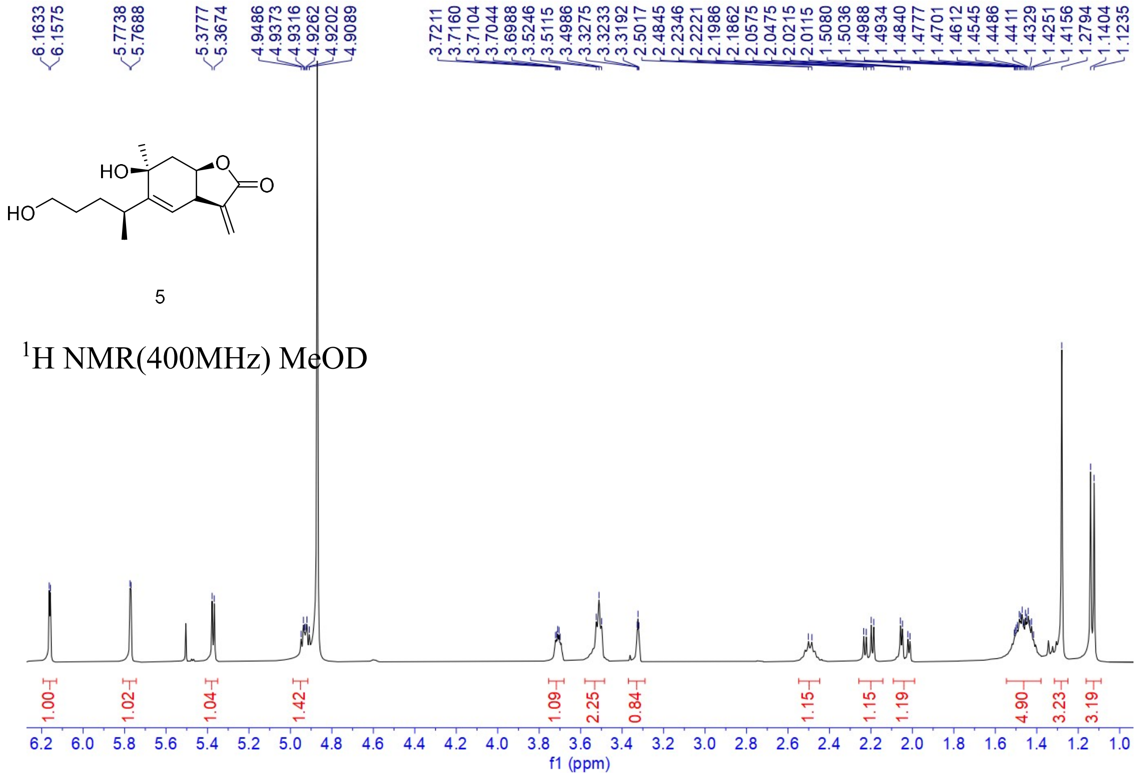


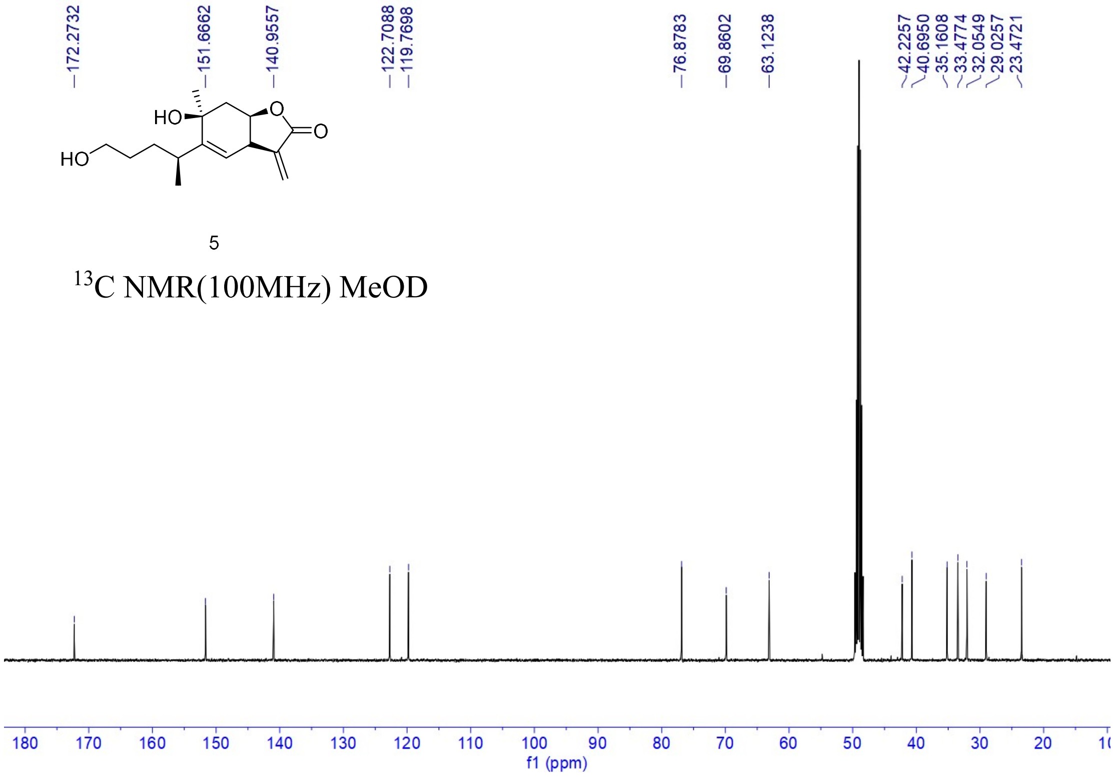

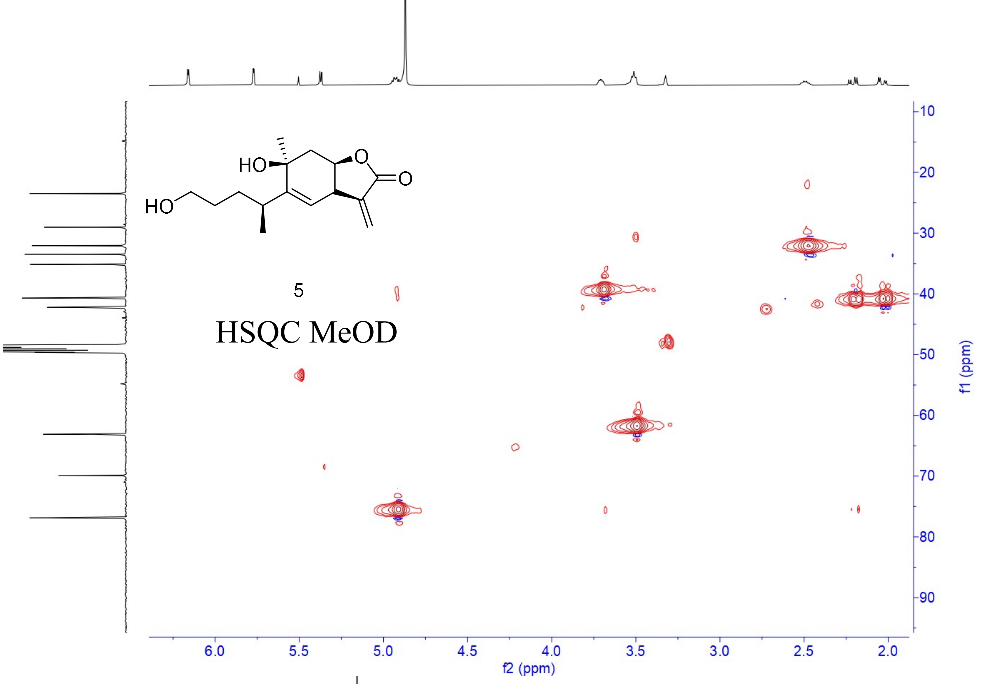
HO
HO
O
O
5
HSQC MeOD
f1 (ppm)
f2 (ppm)

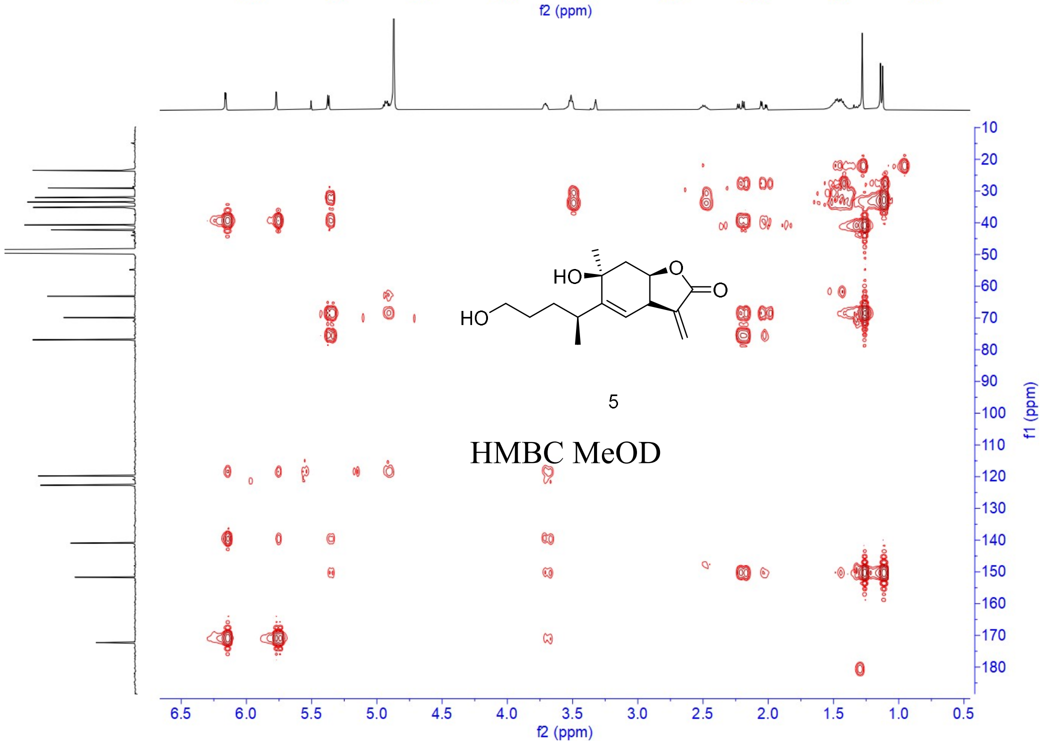
HO
HO
O
O
5
HMBC MeOD
f1 (ppm)
f2 (ppm)

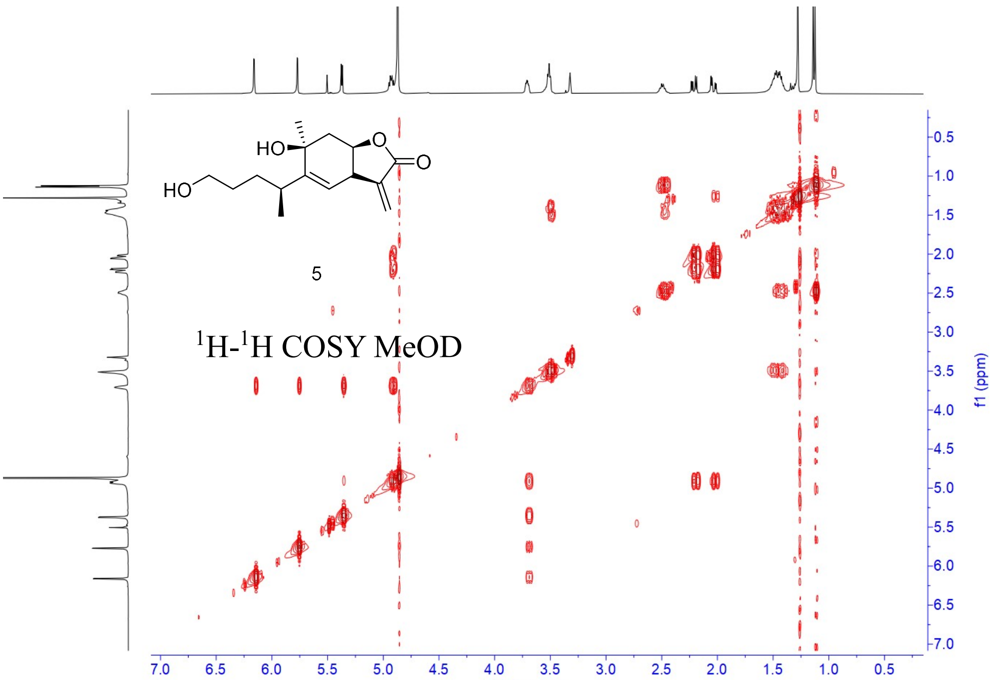
HO
HO
O
O
5
$^{1}$H-$^{1}$H COSY MeOD
f1 (ppm)
f2 (ppm)

**Figure S6.** $^{1}H$ ,$^{13}C$, HSQC, HMBC, $^{1}H$-$^{1}H$ COSY and NOE (MeOD) spectra of **6**

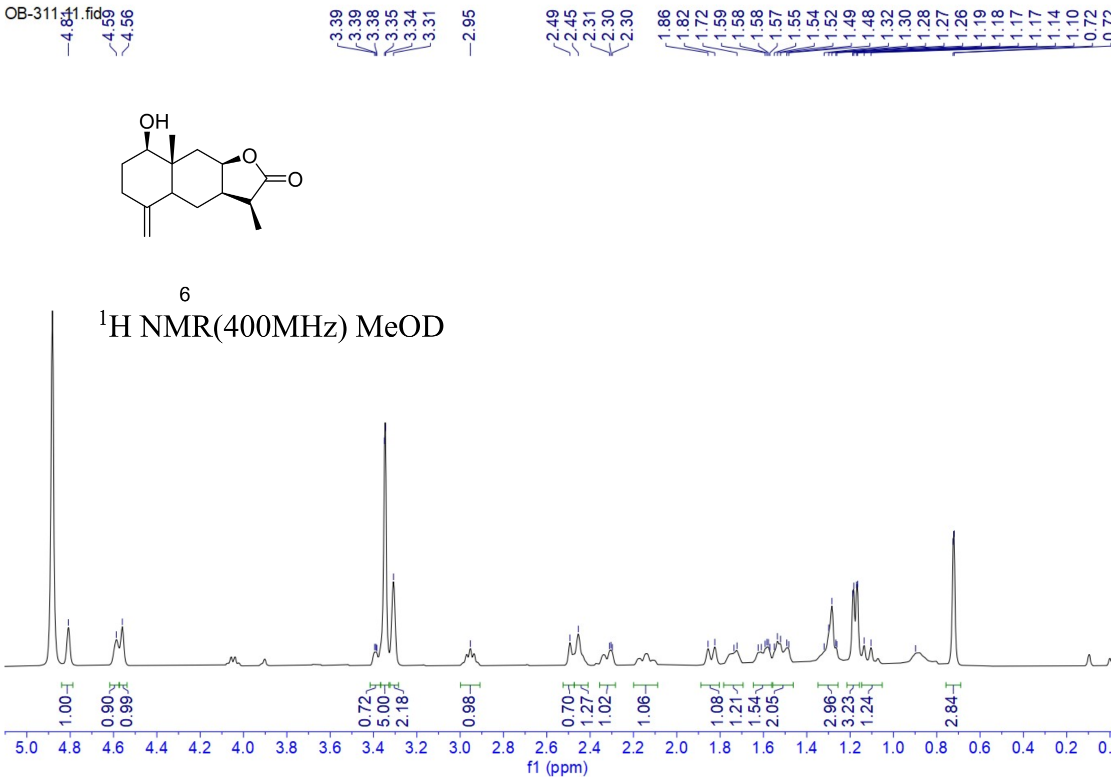


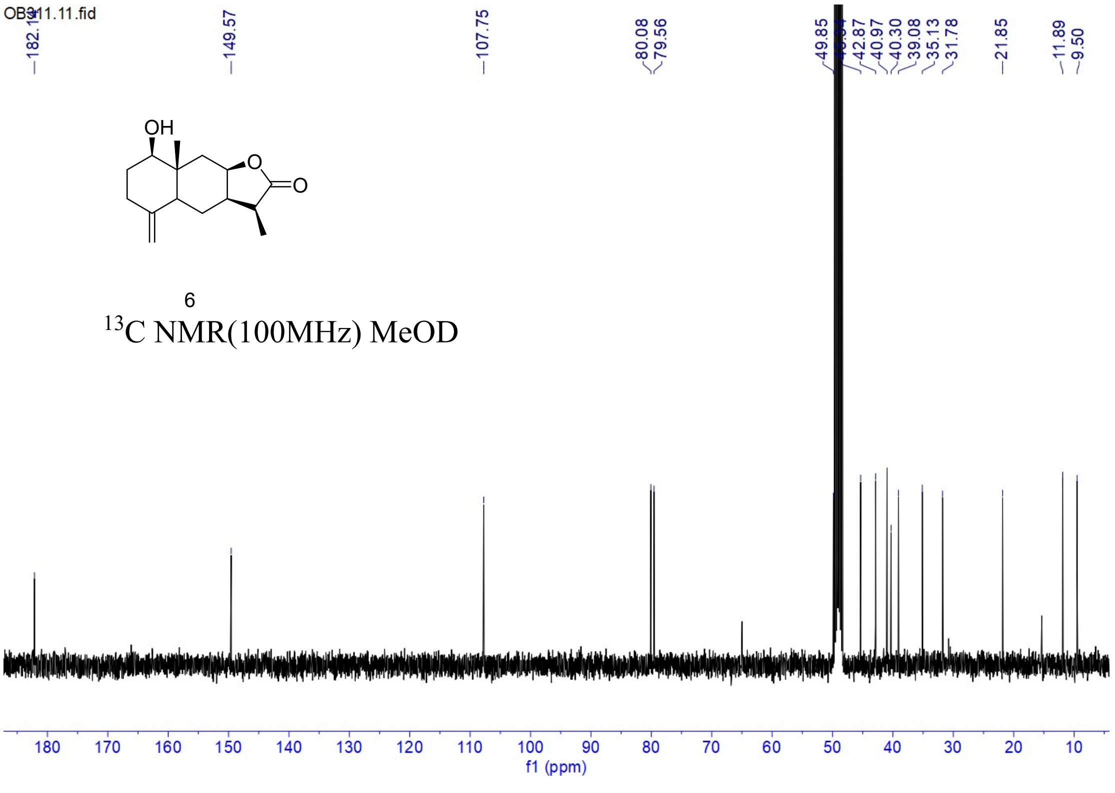

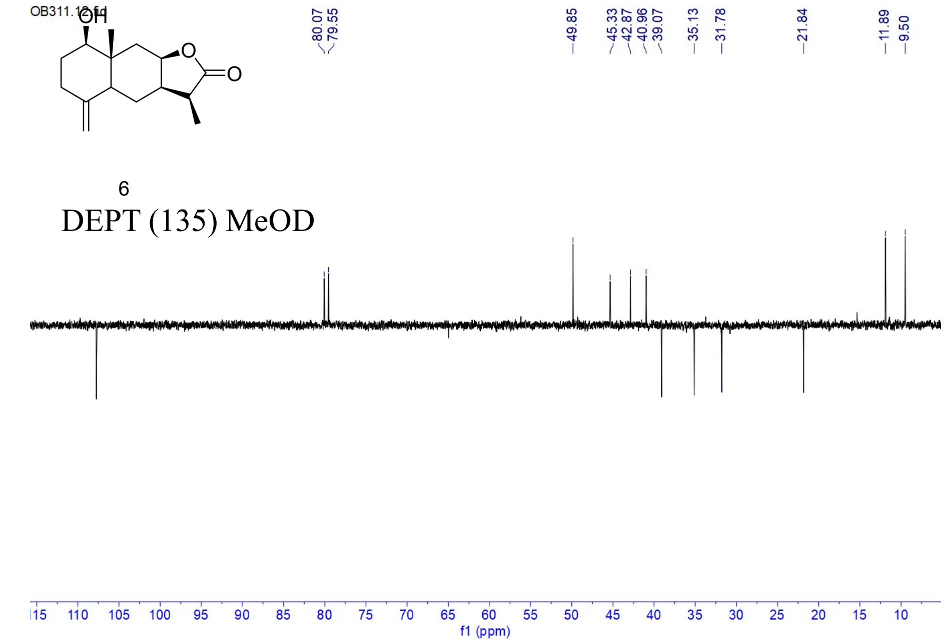
OB311.12.fid
OH
O
O
80.07
79.55
49.85
45.33
42.87
40.96
39.07
35.13
31.78
21.84
11.89
9.50
6
DEPT (135) MeOD
f1 (ppm)

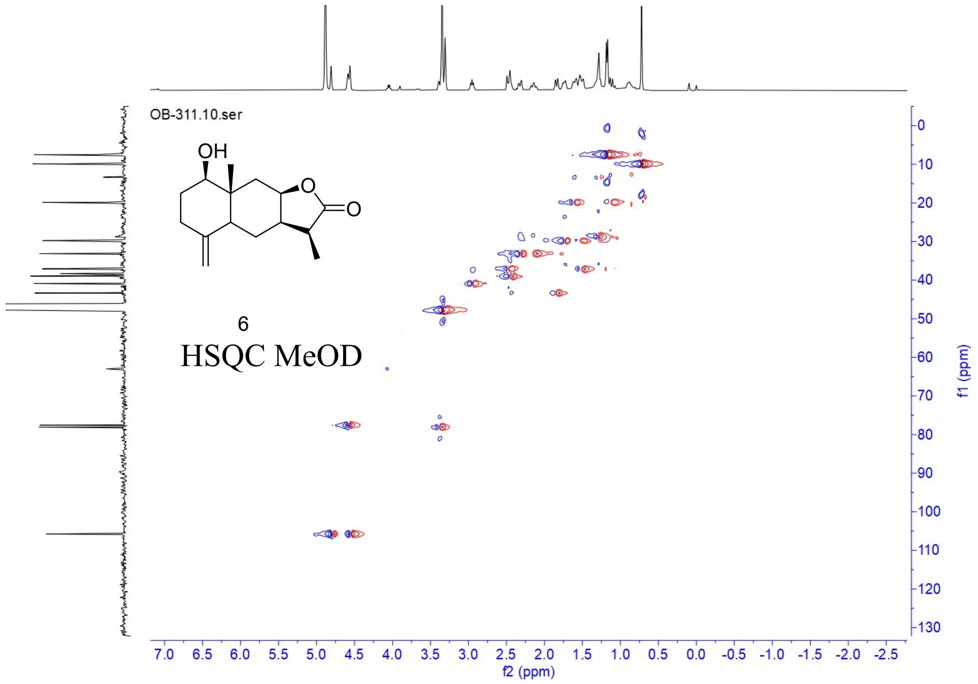
OB-311.10.ser
OH
O
O
6
HSQC MeOD
f2 (ppm)
f1 (ppm)

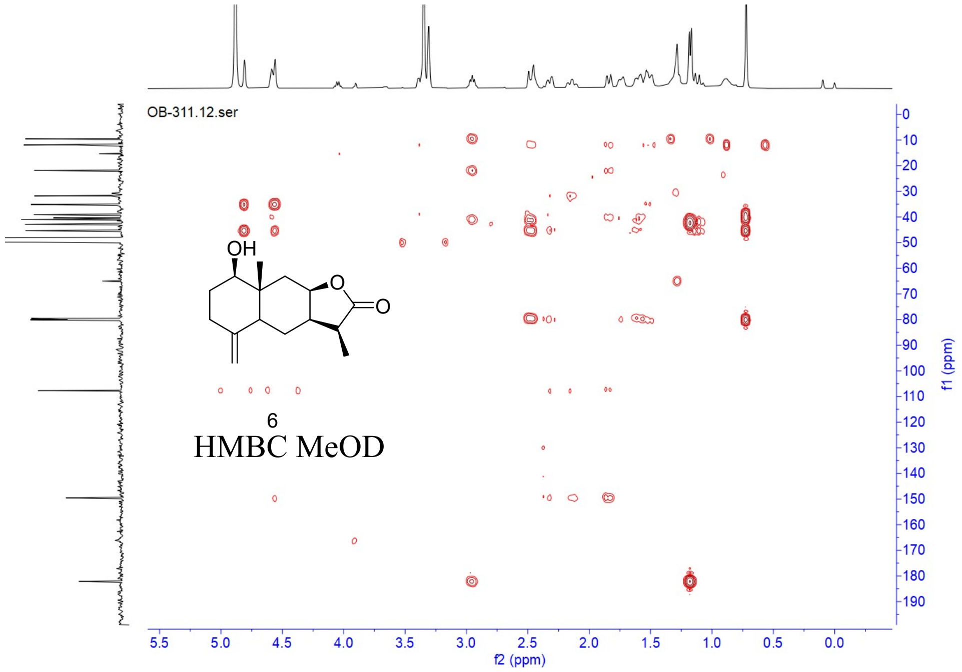
OB-311.12.ser
OH
O
O
6
HMBC MeOD
f2 (ppm)
f1 (ppm)

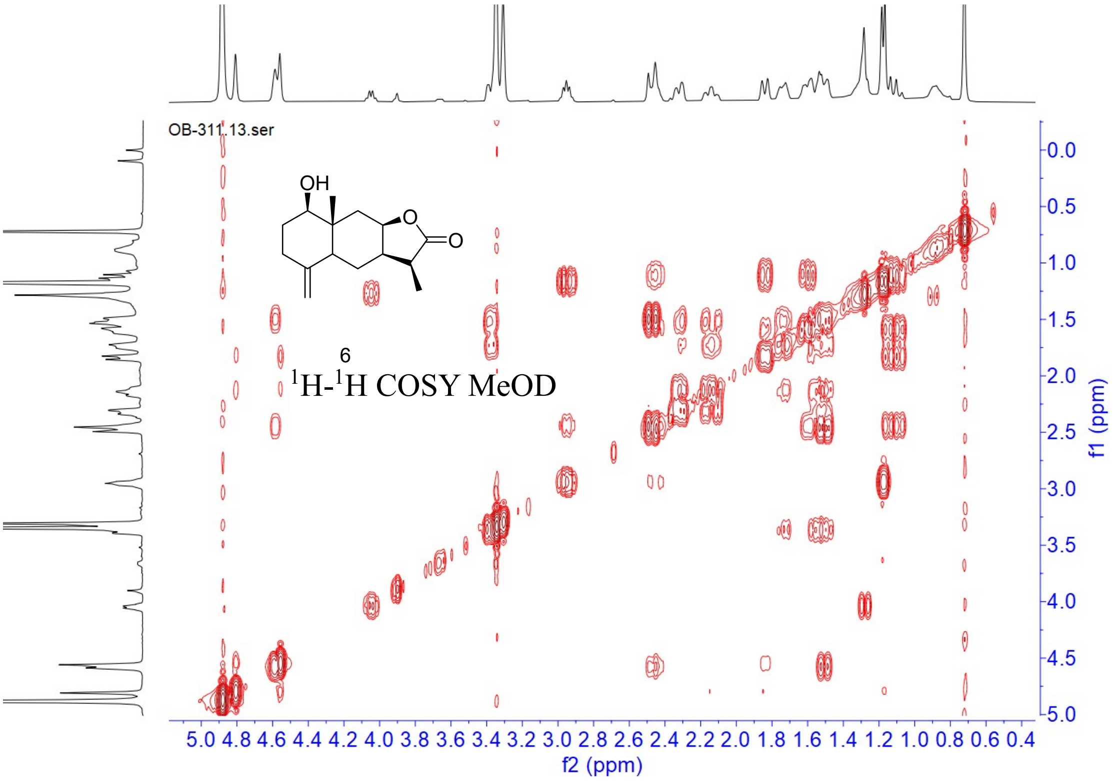
OB-311.13.ser
OH
O
O
6
$^1$H-$^1$H COSY MeOD
f1 (ppm)
f2 (ppm)

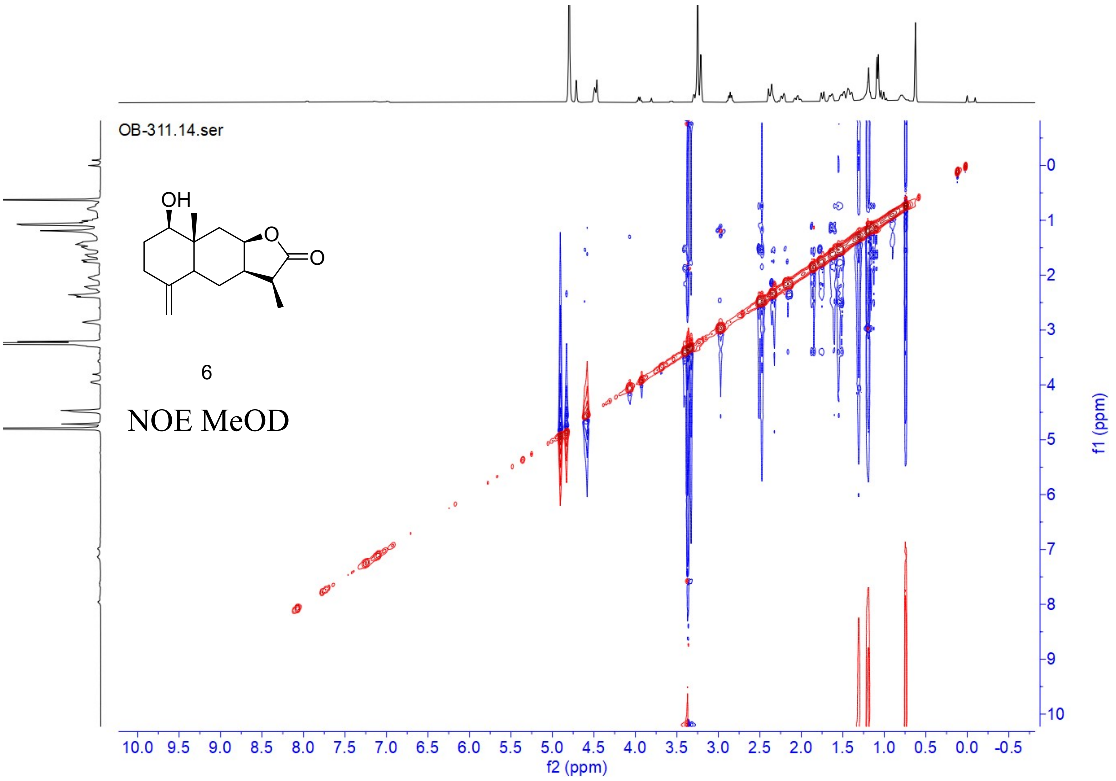
OB-311.14.ser
OH
O
O
6
NOE MeOD
f1 (ppm)
f2 (ppm)

**Figure S7.** $^{1}H$ ,$^{13}C$, HSQC, HMBC and $^{1}H$-$^{1}H$ COSY NMR ($CDCl_3$) spectra of **7**

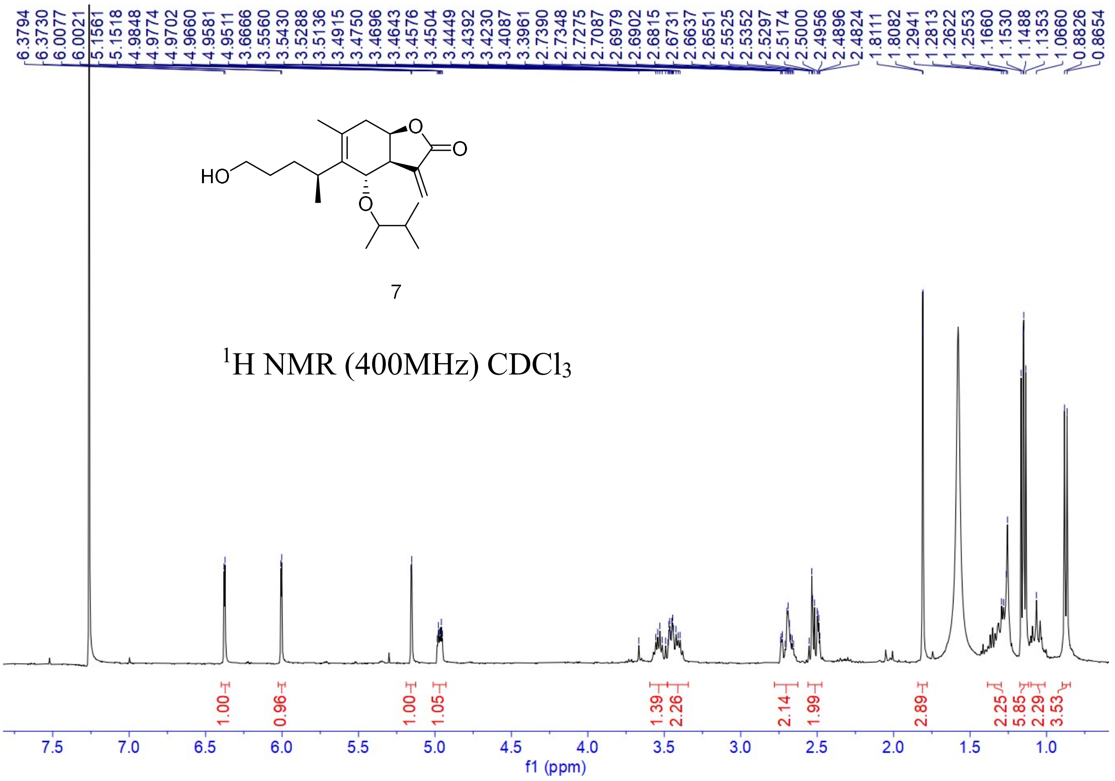


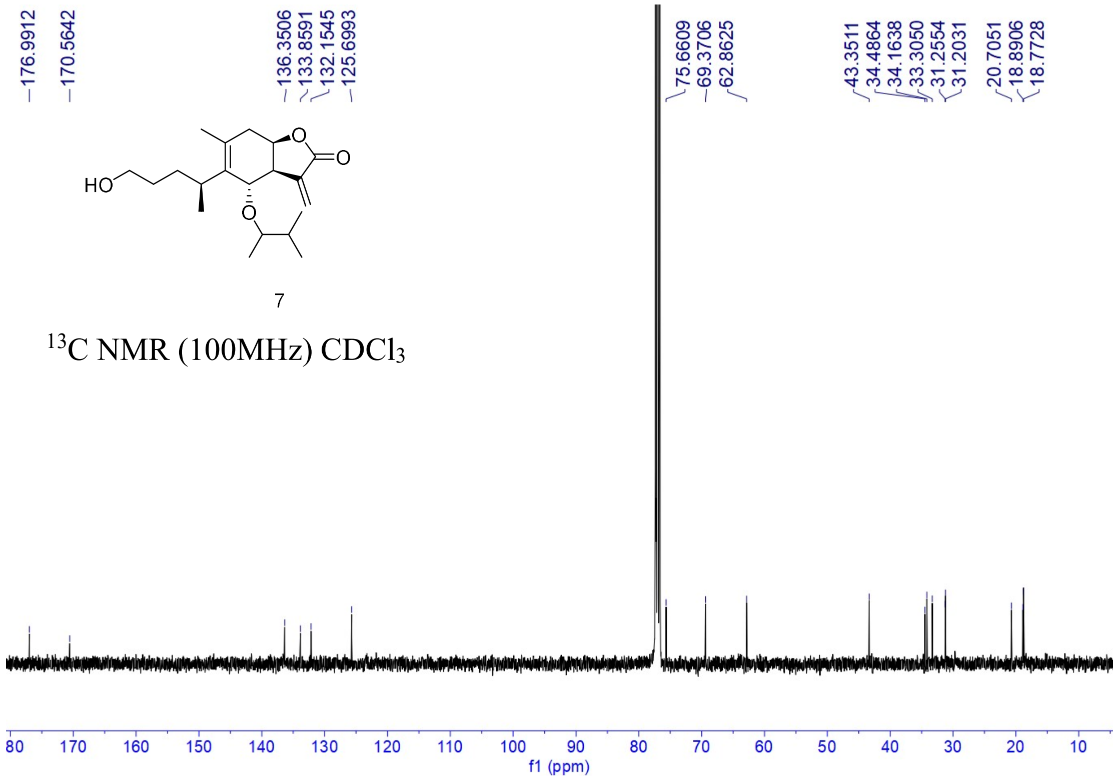

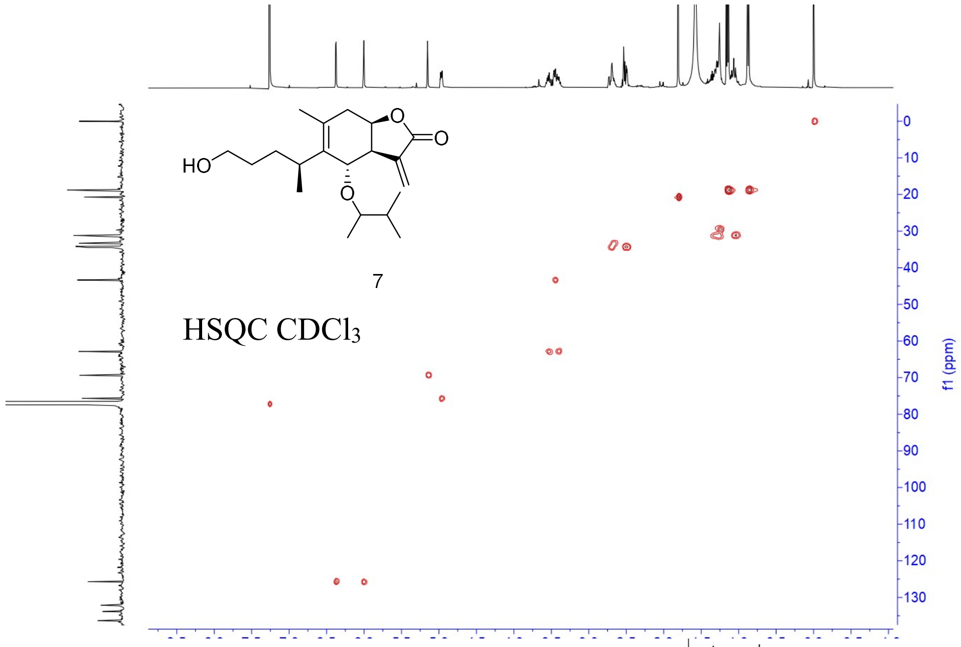
HO
O
O
O
7
HSQC CDCl3
f1 (ppm)

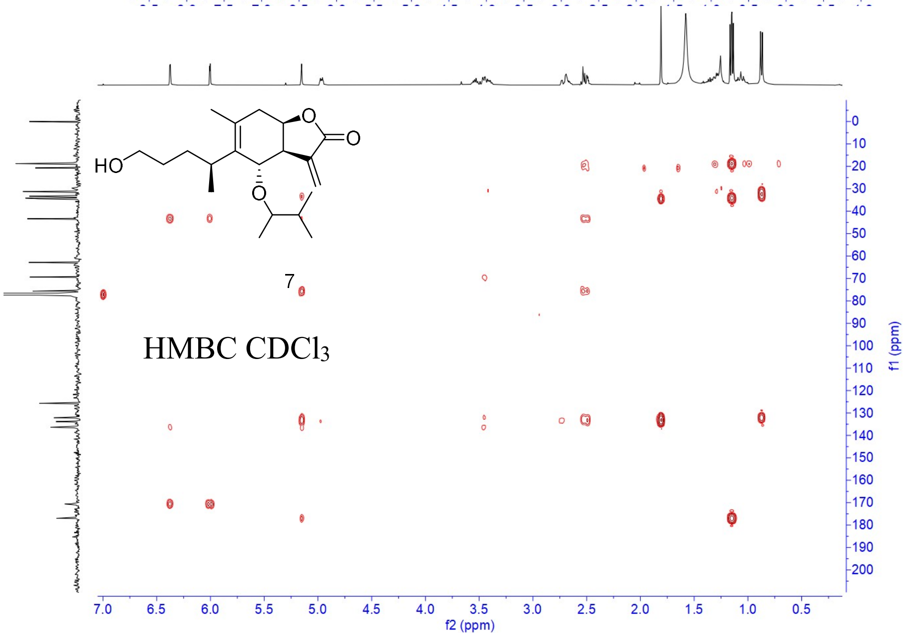
HO
O
O
O
7
HMBC CDCl3
f2 (ppm)
f1 (ppm)

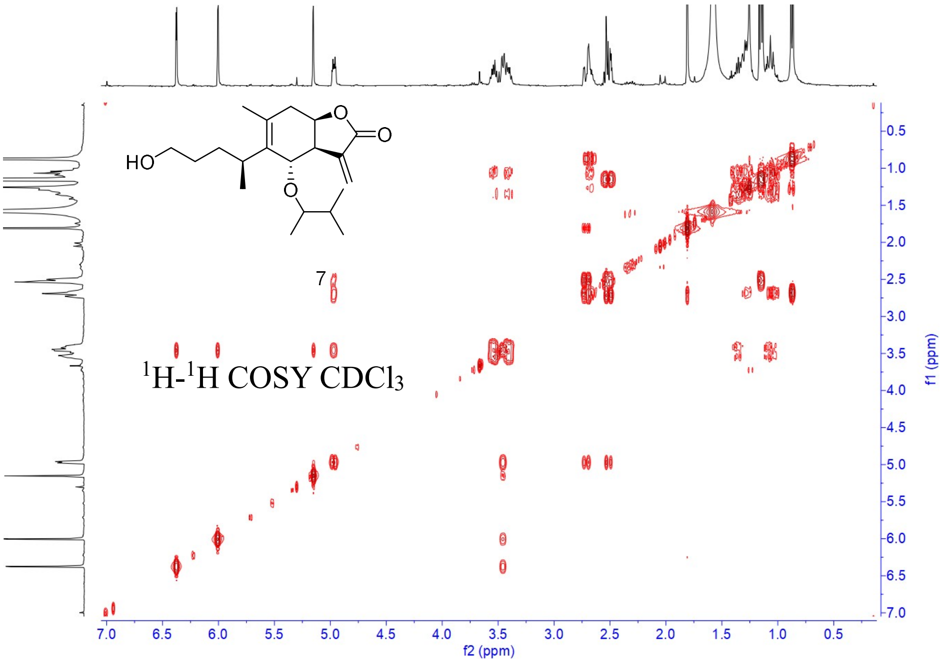
HO
O
O
O
7
1H-1H COSY CDCl3
f2 (ppm)
f1 (ppm)

**FigureS8.** $^{1}$H ,$^{13}$C, HSQC, HMBC and $^{1}$H-$^{1}$H COSY NMR (MeOD) spectra of **8**

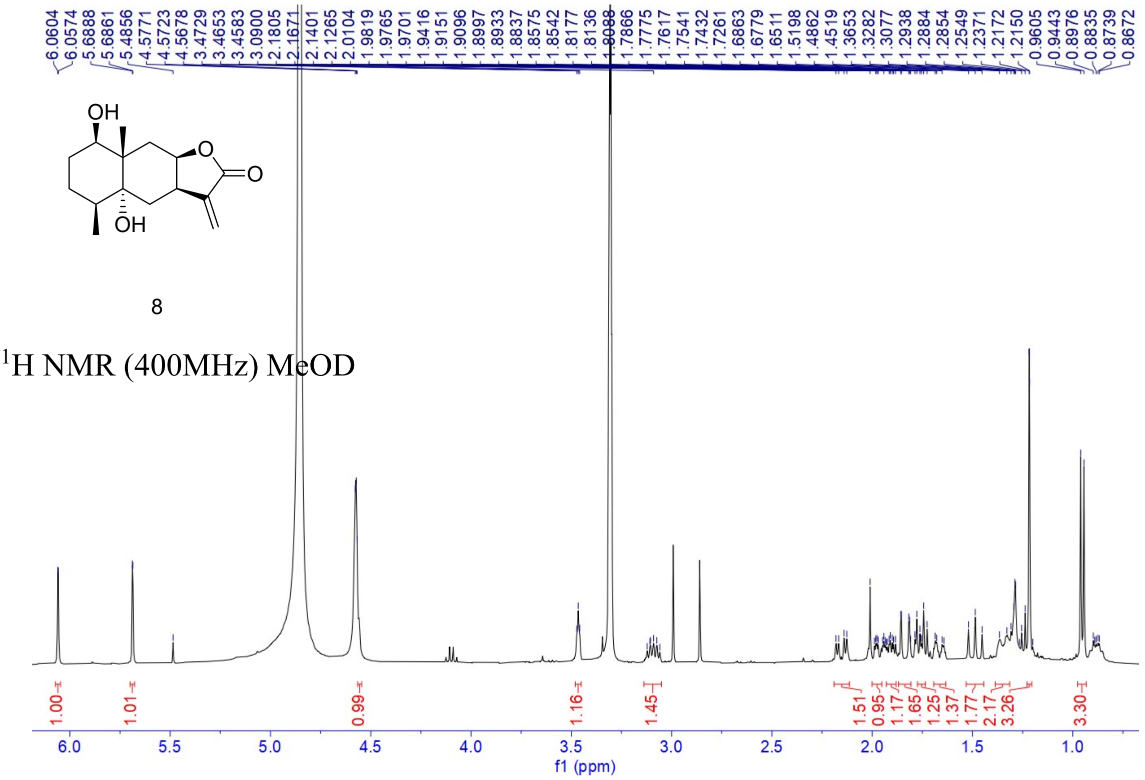


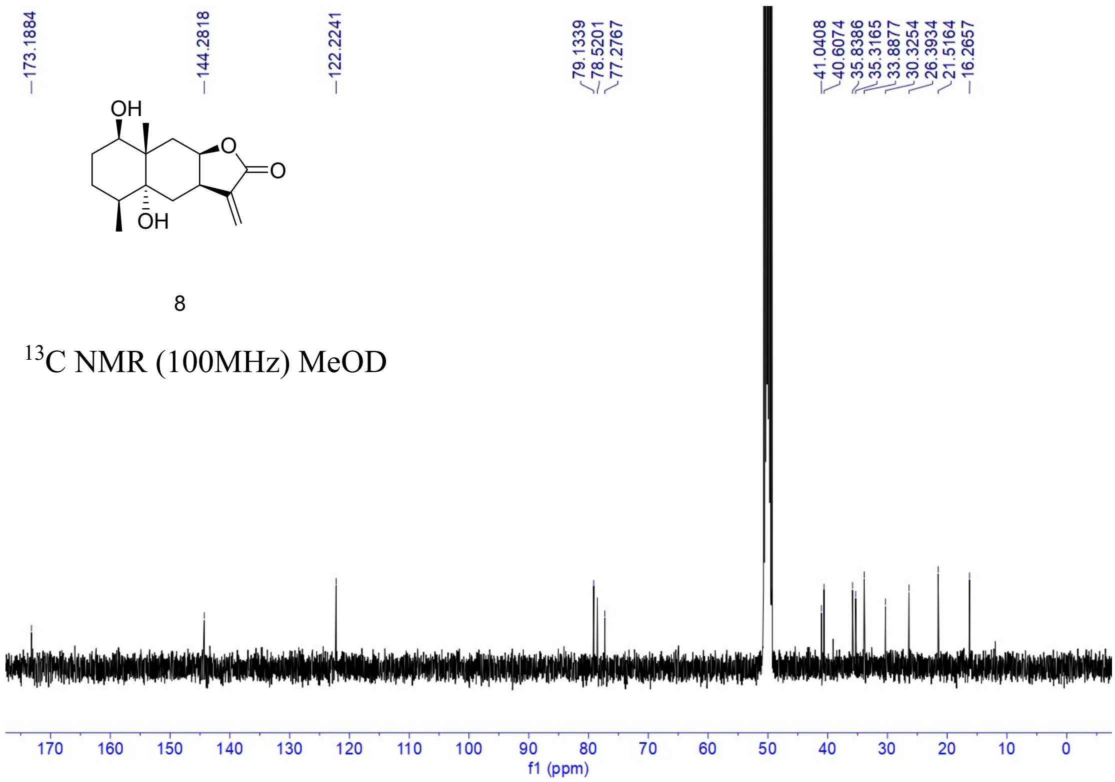

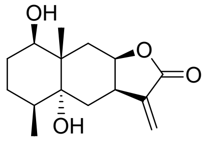

8

DEPT (135) MeOD

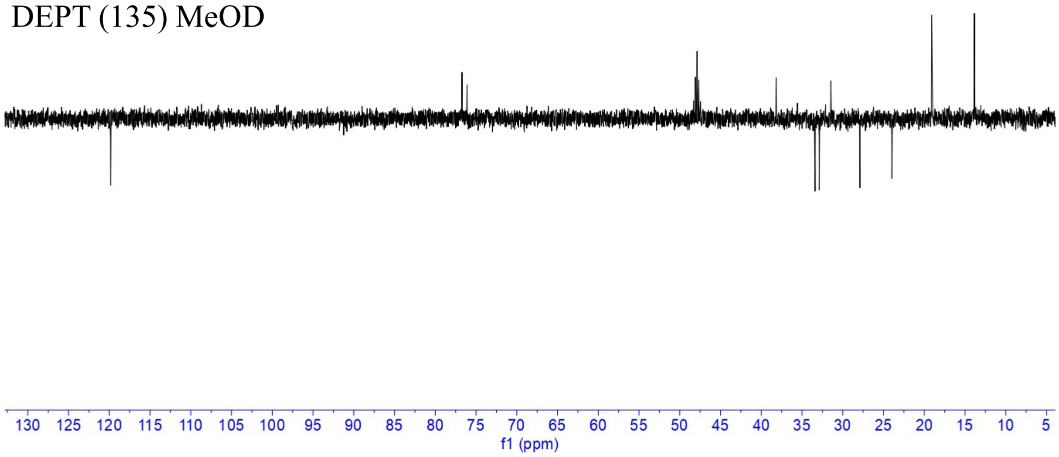


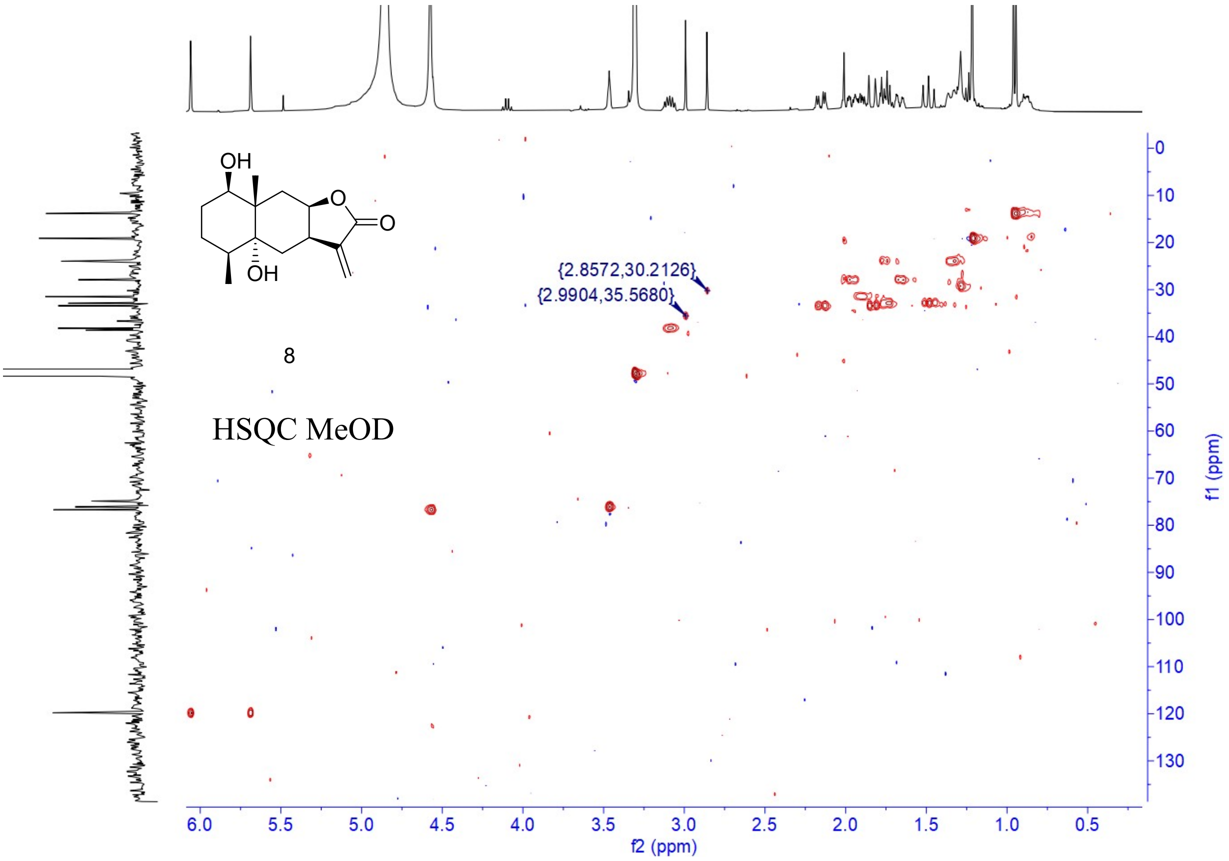

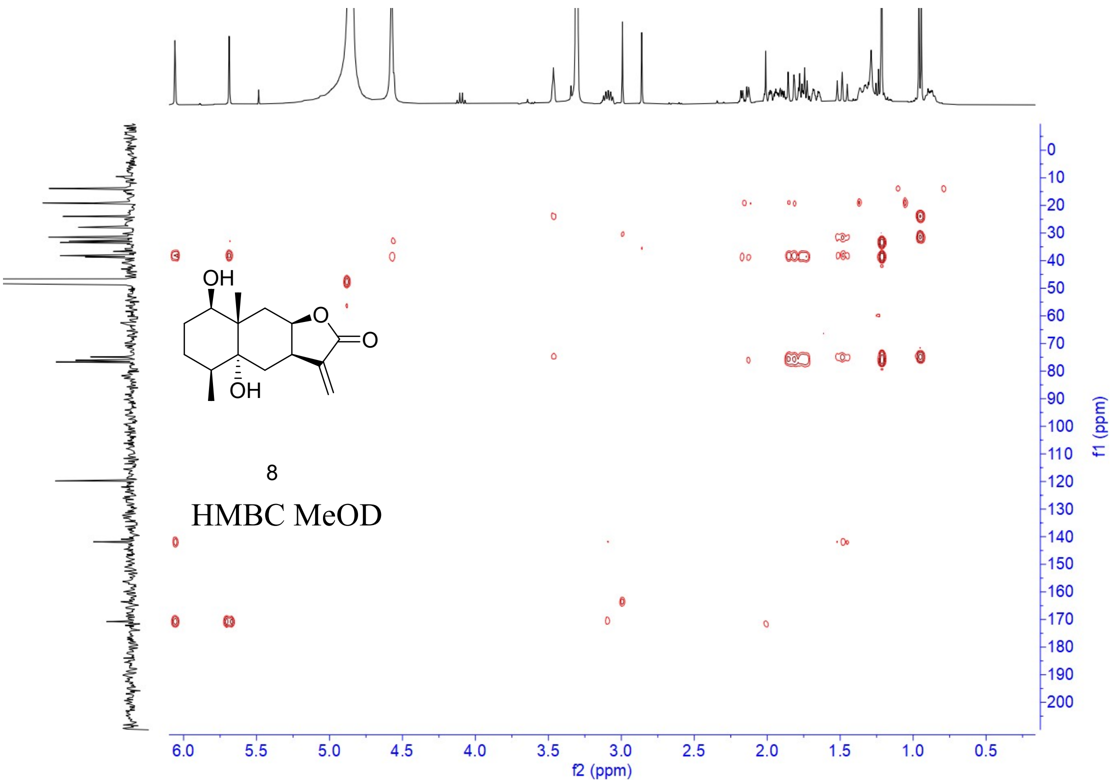
OH
O
O
OH
8
HMBC MeOD
f1 (ppm)
f2 (ppm)

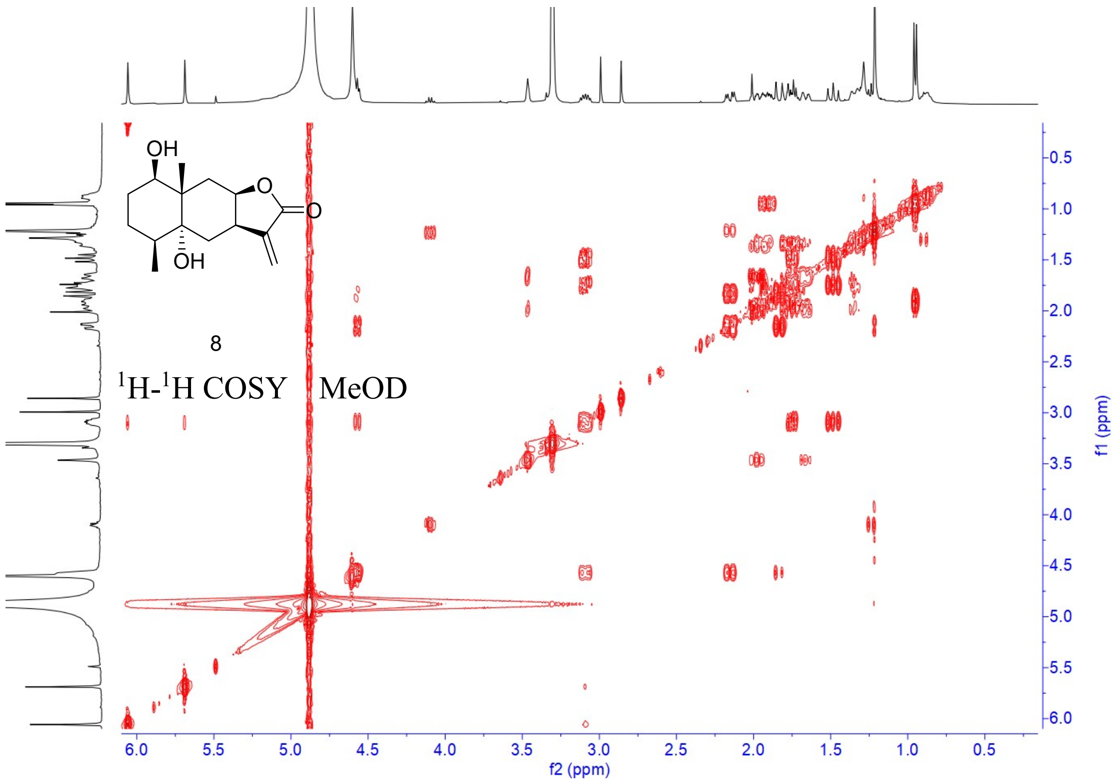
OH
O
O
OH
8
1H-1H COSY
MeOD
f1 (ppm)
f2 (ppm)
